# HYTANE-Identified Latrophilin-3 Cleavage by Meprin β Leads to Loss of the Interaction Domains

**DOI:** 10.1021/acs.jproteome.4c00912

**Published:** 2025-03-26

**Authors:** Fred Armbrust, Kira Bickenbach, Tomas Koudelka, Corentin Joos, Maximilian Keller, Andreas Tholey, Claus U. Pietrzik, Christoph Becker-Pauly

**Affiliations:** † Biochemical Institute, Unit for Degradomics of the Protease Web, University of Kiel, 24118 Kiel, Germany; ‡ Systematic Proteomics & Bioanalytics, Institute for Experimental Medicine, University of Kiel, 24105 Kiel, Germany; § Institute for Pathobiochemistry, University Medical Center of the Johannes Gutenberg University Mainz, 55128 Mainz, Germany

**Keywords:** ADHD, astrocytes, FLRT proteins, N-terminomics, latrophilin-3, meprin β, synapse formation, teneurin

## Abstract

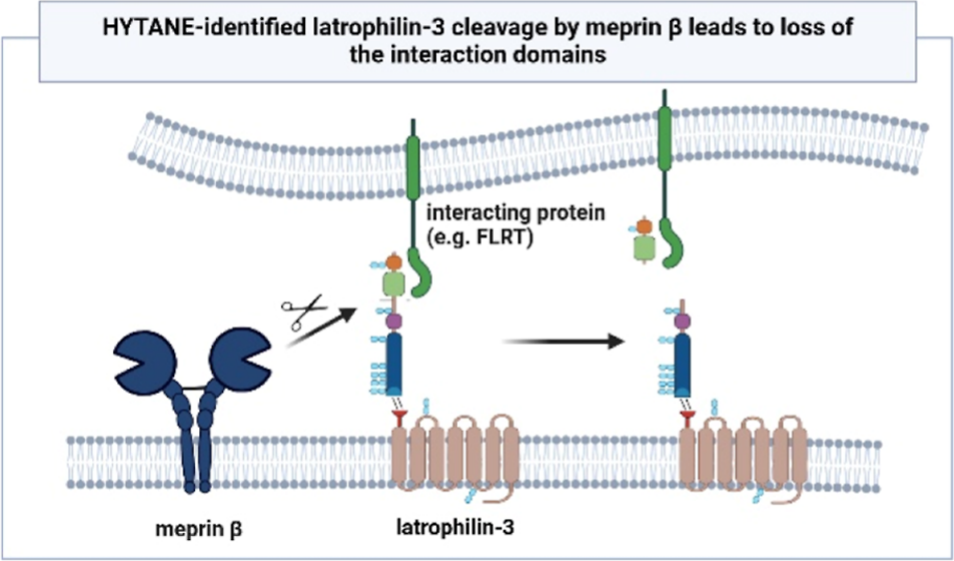

The metalloprotease meprin β is upregulated in neurons and astrocytes of Alzheimer’s disease patients’ brains. While the role of meprin β as the β-secretase of amyloid precursor protein (APP) has been characterized, its broader substrate profile within the brain remains largely unexplored. Hence, to identify additional substrates, we conducted N-terminomics of brain lysates from mice overexpressing meprin β in astrocytes employing the Hydrophobic Tagging-Assisted N-terminal Enrichment (HYTANE) strategy. We observed 3906 (82.2%) N-terminal peptides and identified seven new substrates that match meprin β in terms of localization and cleavage specificity. Of note, the meprin β overexpressing mice show mild cognitive impairments caused by amyloidogenic APP processing alongside hyperactivity and altered exploratory behavior seemingly independent of APP cleavage. Hence, latrophilin-3 was of particular interest, as latrophilin-3 defects are associated with hyperactivity in mice and human. In brain lysates from mice overexpressing meprin β in astrocytes as well as in cellulo, we validated the cleavage of latrophilin-3, resulting in the release of two N-terminal domains. These domains promote interactions with neuronal proteins such as fibronectin leucine-rich repeat transmembrane proteins, promoting adequate synapse formation. Thus, meprin β might affect synaptic integrity by cleaving interaction domains of latrophilin-3, potentially exacerbating the observed hyperactivity phenotype.

## Introduction

The metalloprotease meprin β is upregulated in the brains of Alzheimer’s disease (AD) patients and contributes to the formation of neurotoxic plaques.^1−3^ These plaques primarily consist of amyloid-β (Aβ) peptides, which are generated by the cleavage of the amyloid precursor protein (APP).^4^ Amyloidogenic APP processing predominantly occurs around D672 (according to APP770 numbering) by β-secretases such as BACE1 and meprin β.^1^ Following β-secretase cleavage, the remaining C-terminal fragment (CTF) undergoes further intramembranous processing by the γ-secretase complex, releasing the Aβ peptide into the extracellular space. Its hydrophobic properties promote aggregation into neurotoxic oligomers and plaque deposits.^4^

Our previous studies have underscored the significance of meprin β in AD pathogenesis, demonstrating that its deficiency leads to reduced Aβ release and plaque formation, accompanied by improved memory deficits in an AD mouse model.^3^ Conversely, the pathological overexpression of meprin β in astrocytes has been associated with elevated Aβ levels and mild cognitive deficits including impaired spatial memory, but also altered exploratory behavior and hyperactivity.^5^ Given that hyperactivity and altered explorative behavior are not necessarily a direct consequence of APP cleavage, we aimed to identify novel potential astrocytic substrates of meprin β.

Therefore, in this study, we employed an unbiased N-terminomics approach using the Hydrophobic Tagging-Assisted N-termini Enrichment (HYTANE) strategy on brain lysates from mice overexpressing meprin β in astrocytes compared to control mice. This approach uncovered novel meprin β substrates and identified latrophilin-3 (also named Adhesion G protein-coupled receptor (GPCR) L3) as the most promising candidate. Adgrl3 (the corresponding gene name for latrophilin-3) knockout mice exhibit hyperactivity and an increased susceptibility to addiction.^6^ Furthermore, several studies showed the association of ADGRL3-related single nucleotide polymorphisms with attention-deficit/hyperactivity disorder (ADHD) in humans.^7−9^ Latrophilin-3 is a GPCR expressed in various tissues, e.g., brain and pancreas. In the pancreas, it contributes to insulin secretion by decreasing cyclic adenosine monophosphate (cAMP) levels through its G_i_ activity.^10^ While the brain-expressed splice variant of latrophilin-3 lacks the ability to induce signaling, it plays a pivotal role for synapse formation in brain development. Specifically, latrophilin-3 is expressed on radial glial cells and interacts with proteins on neurons, such as fibronectin leucine-rich repeat transmembrane (FLRT) proteins and teneurins. These interactions occur through latrophilin-3′s N-terminal sea urchin egg lectin (SUEL)-type lectin and olfactomedin-like domains,^11,12^ which is essential for ensuring proper neuron migration and contributing to the development of a functional neuronal network.

## Experimental Section

### Cell Culture and transfection

HEK 293T cells were maintained at 37 °C under an atmosphere of 5% CO_2_ in Dulbeccó’s modified Eagles medium (DMEM; Thermo Fisher Scientific) supplemented with 10% fetal bovine serum (FBS; Thermo Fisher Scientific). Transfection with plasmid-DNA, premixed with polyethylenimine (PEI) (1:3) in serum-free medium, was performed at 80–90% cell confluence. Plasmid DNA coding for HA tagged murine latrophilin-3 wildtype (wt) or latrophilin-3 variants with amino acid exchanges around the cleavage site, human wildtype (wt) meprin β, and the human catalytically inactive meprin β variant E153A, human meprin β T324A variant, the human meprin α, the human catalytically inactive meprin α variant E156A, human ADAM10, the human catalytically inactive ADAM10 variant E384A, human ADAM17, human BACE1, human MT1-MMP, the human catalytically inactive MT1-MMP variant E240Aand pcDNA3.1 as empty vector control in different combinations were added together with transfection reagent to the cell culture medium. After 24 h, the cell medium was changed to serum-free DMEM, as serum components inhibit meprin β activity.

### Site-Directed Mutagenesis

To generate plasmids coding for human meprin β T324A, and latrophilin-3 variants with amino acid exchanges at or close to the cleavage site of meprin β, site-directed mutagenesis was used to exchange individual nucleotides. In this approach, PCRs were conducted using Phusion High-Fidelity DNA Polymerase (Thermo Fisher Scientific) and the following oligonucleotide primers, which were beforehand phosphorylated using T4 Polynukleotide Kinase (Thermo Fisher Scientific) according to the manufacturer’s instructions: meprin β T324A for: TGTGGGGGCCGCGGCAGTGCTGG; meprin β T324A rev: TTTACAGAGCTGCTATCGAAATGCATGAAGAAACCAG; latrophilin-3 D484A for: AATTCACCTCGCGTCTGAACTAGAAAG; latrophilin-3 D484A rev: GGTGGAGAGATGTAGG; latrophilin-3 E486 for: CCTCGACTCTGCGCTAGAAAGGC; latrophilin-3 E486 rev: TGAATTGGTGGAGAGATG; latrophilin-3 E488 for: TCGAGGTGAATTGGTGG; latrophilin-3 E4846 rev: GCGAGGCCCCCTGTCAGAG; latrophilin-3 484-488→A for: CGGCGGCGAGGCCCCCTGTCAGAG; latrophilin-3 484-488→A rev: CCGCCGCGAGGTGAATTGGTGGAGAG (exchange of amino acids 484 to 488 to alanine). Afterward, to eliminate template DNA, the samples were incubated with DpnI restriction endonuclease (Thermo Fisher Scientific) according to the manufacturer’s instruction. Subsequently, the plasmids were heat-shock transformed in*Escherichia coli* XL1 blue chemically competent bacteria for amplification, followed by plasmid purification. The sequence of the obtained plasmid was confirmed by Sanger sequencing (Eurofins Genomics).

### Experimental Animals

Mice were maintained under a 12 h light/12 h dark cycle with access to water and standard mouse diet *ad libitum* in individually ventilated cages in accordance with the ethical standards set by the National Animal Care Committee of Germany. Meprin β knock-in mice (Rosa26^Mep1b–HA^) expressing meprin β with C-terminal HA tag under the control of the glial fibrillary acidic protein promoter in astrocytes were generated as described in.^5^ Rosa26^*Mep1b–HA*^ mice, which were heterozygous for GFAP^Cre^, are termed GFAP^Cre+/–^;Rosa26^*Mep1b*–HA^ mice (or briefly GFAP^Cre+/–^). The respective Cre-negative control animals are termed GFAP^Cre–/–^;Rosa26^*Mep1b*–HA^ (or briefly GFAP^Cre–/–^). To generate mice overexpressing meprin β with C-terminal HA tag in neurons of the hippocampus and cortex, meprin β knock-in mice (Rosa26^Mep1b–HA^) were crossed with NEX^Cre^ mice.^13^ Rosa26^*Mep1b–HA*^ mice, which were heterozygous for NEX^Cre^, are termed NEX^Cre+/–^;Rosa26^*Mep1b*–HA^ mice (or briefly NEX^Cre+/–^). The respective Cre-negative control animals are referred to as NEX^Cre–/–^;Rosa26^*Mep1b*–HA^ (or briefly NEX^Cre–/–^).

### Generation of Mouse Brain Lysates

Mice were sacrificed by cervical dislocation in accordance with the Guide for the Care and Use of Laboratory Animals (German Animal Welfare Act on Protection of Animals), and brains were isolated. For whole brain lysates, the brains were homogenized in triton lysis buffer (1% (v/v) Triton X-100 (Roth), cOmplete protease inhibitor cocktail (Roche) in PBS) using the Precellys 24 (VWR) for 3 cycles at 3000 rpm. The homogenates were incubated for 1 h at 4 °C. The debris was removed by centrifugation for 15 min at 16,000 g and 4 °C.

### Generation, Cultivation and Lysis of Organotypic Brain Slice Cultures

To generate OBSCs, mice were sacrificed by cervical dislocation in accordance with the Guide for the Care and Use of Laboratory Animals (German Animal Welfare Act on the Protection of Animals). The procedure was previously described in ref (5). In brief, the brain was sagitally cut in the middle of one hemisphere with a razorblade. The cut side was subsequently glued to the specimen plate of a vibratome (VT1200S, Leica). Using the vibratome, 250 μm sagittal OBSCs were generated at a speed of 0.03 mm/s and an amplitude of 3 mm. The OBSCs were cultivated for 19 days in a serum-containing medium. In order to avoid meprin β inhibition by serum components, the OBSC culture medium was substituted by serum-free OBSC medium for 24 h before harvest. For tissue lysis, OBSCs were transferred into reaction tubes and incubated for 1 h at 4 °C in triton lysis buffer. The debris was removed by centrifugation for 15 min at 16,000*g* and 4 °C.

### Synaptosome Isolation

Animals were sacrificed by cervical dislocation, and brains were removed. For further characterization, brains were dissected into the cortex and hippocampus. To obtain enough protein, three hippocampi from each group were pooled. Functional synaptosomes were isolated as recommended by the manufacturer (ThermoFisher, no. 87793). Briefly, brain regions were weighed and mechanically homogenized in an appropriate amount of the Syn-PER reagent. First, the homogenate was centrifuged at 1200*g* for 10 min. The supernatant was transferred to a fresh tube. After centrifugation at 15,000*g* for 20 min, the pellet was resuspended in Syn-PER reagent, which was supplemented with cOmplete protease inhibitor cocktail (Roche), to a final protein concentration of 4–5 μg/μL, resulting in the synaptosome fraction. The synaptosome fraction was aliquoted and snap frozen for further analysis.

### Cell Harvest and Lysis

For SDS–polyacrylamide gel electrophoresis (PAGE) and Western blot analyses, cells were harvested 24 h after the medium change to serum-free DMEM, to avoid inhibition of meprin β by serum components. The supernatant was removed, and cells were washed with PBS and then incubated in lysis buffer (1% (v/v) Triton X-100, cOmplete protease inhibitor cocktail (Roche) in PBS (pH 7.4)) for 30 min at 4 °C. The lysates were centrifuged for 15 min at 15,000*g* and 4 °C, and the cell debris was discarded. Protein concentration in the lysates was determined using the Pierce BCA Protein Assay Kit (Thermo Fisher Scientific) according to the manufacturer’s instructions. Cell supernatants were ultracentrifuged for 2 h at 186,000*g* and 4 °C. To concentrate soluble proteins from cell supernatants, a trichloroacetic acid (TCA) precipitation was performed. For this, supernatants were mixed with TCA at a final concentration of 10% (v/v) TCA and incubated for 30 min on ice. Afterward, supernatants were centrifuged for 20 min at 15,000*g* and 4 °C; supernatants were discarded, and pellets were carefully washed with precooled acetone (−20 °C). After centrifugation for 15 min at 15,000*g* and 4 °C, the acetone was discarded and evaporated completely. Precipitated proteins from supernatants as well as cell lysates were then dissolved in the sample buffer, heated for 10 min at 95 °C, and analyzed by SDS-PAGE and Western blot analysis.

### Cell Surface Biotinylation Assay

For the cell surface biotinylation assay, cultivated cells were washed with PBS-CM (0.1 mM calcium chloride, 1 mM magnesium chloride in PBS) and incubated with biotin solution [1 mg/mL Sulfo-NHS-SS-Biotin (Thermo Fisher Scientific)] in PBS-CM. Subsequently, cells were incubated with quenching buffer (50 mM Tris–HCl, pH 8.0 in PBS-CM) for 10 min. Afterward, cells were lysed in 450 μL biotinylation lysis buffer [50 mM Tris–HCl (Roth) (pH 7.4), 150 mM NaCl (Roth), 1% (v/v) Triton-X 100 (Roth), 0.1% (w/v) sodium dodecyl sulfate (SDS) (Roth), cOmplete protease inhibitor cocktail (Roche)] for 30 min. 50 μL was separated as a lysate control. Pierce magnetic streptavidin beads (Thermo Fisher Scientific) were added to the remaining lysate and the mixture incubated for 1 h. Afterward, the beads were washed with biotinylation lysis buffer, and the proteins were removed and denatured by addition of sample buffer for 10 min at 95 °C. Afterward, the samples were analyzed by SDS-PAGE and Western blot.

### Immunoprecipitation of Latrophilin-3 Cleavage Fragments from Cell Supernatants

For immunoprecipitation of latrophilin-3 cleavage fragments, cells were harvested 24 h after the medium change to serum-free DMEM. The supernatant was removed and ultracentrifuged for 2 h at 186,000*g* and 4 °C. Immunoprecipitation of latrophilin-3 cleavage fragments from ultrazentrifuged cell supernatant was performed using HA tag antibody (C29F4; Cell Signaling; 0.3 μg/mL final concentration) and Protein G Dynabeads (Thermo Fisher Scientific) according to the manufacturer’s instructions. Dynabead antibody–antigen complexes were washed with PBS and proteins were denatured by addition of sample buffer for 10 min at 95 °C. Afterward, immunoprecipitated proteins and lysate controls were analyzed by SDS-PAGE and Western blot. For the detection of immunoprecipitated proteins, Clean-Blot IP Detection Reagent (HRP) (Thermo Fisher Scientific) was used.

### Purification of Soluble Meprin β

Soluble wt meprin β and meprin β E153A were purified from serum-free supernatants of HEK 293T cells transfected with N-terminally strep-tagged human wt meprin β or meprin β E153A together with human ADAM10. Supernatants were concentrated using a 10 kDa Amicon column (Merck) by centrifugation at 3500*g* and 4 °C and strep-tagged soluble meprin β were purified with strep-tactin resins gravity flow columns (IBA lifesciences) at 4 °C according to the manufacturer’s instructions. With the eluent, a buffer exchange to 20 mM HEPES (pH 7) was performed using 10 kDa Amicon columns. To activate soluble wt meprin β and meprin β E153A (in a concentration of 100 nM), 10 μg/mL (final concentration) trypsin in HEPES was added, and the samples were incubated at 37 °C for 15 min. Afterward, ovomucoid (25 μg/mL final concentration) was added, and samples were incubated at 37 °C for 15 min. As control, HEPES alone was incubated with trypsin and ovomucoid. Activated soluble wt meprin β and meprin β E153A or the control (HEPES, trypsin, ovomucoid) was given into serum-free supernatant of HEK cells in a final concentration of 5 nM meprin β for 24 h prior to cell harvest.

### SDS-PAGE and Western Blot Analysis

Prior to SDS-PAGE, the protein concentration in all lysates was determined using the BCA protein assay kit (Thermo Fisher Scientific) according to manufacturer’s instructions. Lysates were incubated for 10 min at 95 °C with sample buffer to final concentrations of 50 mM Tris–HCl (pH 6.8), 2% (w/v) SDS, 0.1% (w/v) bromophenol blue (Merck), 10% (v/v) glycerol (Roth), and 30 mg of dithiothreitol (DTT) (Roth). The protein separation was performed by SDS-PAGE (120 V, 90 min) using the Mini-PROTEAN system (Bio-Rad) system. Afterward, Western blotting was continued. The latter was accomplished with a tank-blot system (Bio-Rad), and protein transfer was done onto PVDF membranes (0.8 A, 2 h, 4 °C). The membranes were blocked with 5% (w/v) milk in TBS for 1 h at room temperature. The primary antibodies against meprin β [polyclonal antibody, generated against a peptide from the meprin/A5 protein/receptor protein tyrosine phosphatase μ (MAM) domain (1:1000; Pineda)], HA tag (C29F4; 1:1000; Cell Signaling), glyceraldehyde 3-phosphate dehydrogenase (GAPDH) (14C10; 1:5000; Cell Signaling), latrophilin-3 (B-6; 1:500; Santa Cruz), transferrin receptor 1 (TfR) (ab84036; 1:1000; Abcam), meprin α [polyclonal antibody, generated against peptide from the ectodomain (1:1000, Pineda), ADAM17 (A300D), [generated by Institute of Biochemistry, Kiel University; 1:1000], ADAM10 (AB124695, abcam, 1:1000), BACE1 (A17035K, Biolegend, 1:1000) and MT1-MMP (mab3328, Sigma-Aldrich, 1:1000) were incubated in the indicated dilution with the membrane overnight at 4 °C. Horseradish peroxidase-conjugated secondary antibodies (Jackson ImmunoResearch) were diluted in TBS-T (TBS with 0.1% (v/v) Tween20) and incubated with the membranes for 1 h at room temperature. The chemoluminescence signal was detected in the Amersham ImageQuant 800 (Cytiva) or Intelligent Dark Box (LAS-3000, Fujifilm) using the WesternBright ECL HRP substrate (Advansta) or Super Signal West Pico/Femto Kit (Thermo Fisher Scientific) according to manufacturer’s instructions.

### HYTANE Analysis to Identify New Astrocytic Substrates of Meprin β

In order to identify new meprin β substrates in murine astrocytes with the Hydrophobic Tagging-Assisted N-termini Enrichment strategy (HYTANE), the brains of three GFAP^Cre+/–^;Rosa26^*Mep1b*–HA^ and GFAP^Cre–/–^;Rosa26^*Mep1b*–HA^ mice were isolated and lysed in RIPA lysis buffer [10 mM Tris–HCl (pH 8.0), 1 mM EDTA (Roth), 0.5 mM EGTA (Roth), 1% (v/v) Triton X-100, 0.1% (w/v) sodium deoxycholate, 0.1% (w/v) SDS, 140 mM NaCl)]. The protein concentration was determined using a Pierce BCA Protein Assay Kit (Thermo Fisher Scientific) according to the manufacturer’s instructions. 2.5 mg protein of each sample was precipitated by the addition of a 9-fold volume of ethanol. The pellets were incubated for 2 h at −20 °C and subsequently centrifuged at 16,000*g* for 20 min 300 μL of 6 M guanidine hydrochloride in 100 mM tris(2-carboxyethyl)phosphine (TCEP) was added to the pellets, and these were dissolved on ice with the aid of sonication (5 min). After a centrifugation step at 21,100*g* for 5 min at 4 °C, the supernatants were removed and BCA analysis was performed to determine the protein concentration. 100 μg of each sample was reduced with 5 mM TCEP for 30 min at 65 °C and then alkylated with 12.5 mM iodoacetamide at room temperature for 30 min. The samples were labeled with Tandem Mass Tag reagent in equal volume of dimethyl sulfoxide (DMSO). The samples were left to react for 1 h at 25 °C and then quenched with 8 μL of 5% (m/v) hydroxylamine for 30 min at 37 °C. All channels were combined, and the sample was chloroform/methanol/water precipitated. The pellets were washed with methanol and then redissolved in 3 M guanidine hydrochloride and then diluted to a final concentration of approximately 0.85 M. The sample was digested with trypsin (approximately 50:1 ratio of protein to enzyme) overnight at 37 °C. The sample was cleaned using a C-18 column and eluted with elution buffer [80% (v/v) acetonitrile (ACN) 0.1% (v/v) trifluoracetate (TFA)]. Approximately 60 μg of the sample was used for preHYTANE, while for the rest of the sample, the HYTANE was applied for depletion and the neo-N-termini generated employing trypsin digestion. The samples were dissolved in 200 mM HEPES buffer (pH 7). Then, hexadecanal (500 μL, 10 mg/mL) in isopropanol was added along with 20 mM sodium cyanoborohydride and the reaction left for 4 h at 50 °C followed by 37 °C overnight. 20 mM sodium cyanoborohydride was added, and the sample was dried down. Afterward, it was resuspended in loading buffer (3% ACN, 0.1% TFA) and cleaned with a C-18 column. The sample was dried down and stored at −20 °C prior to analysis.

For the mass spectrometry (MS) analysis, the samples were injected in duplicate on a Dionex Ultimate 3000 nano-UHPLC coupled to a Q Exactive mass spectrometer (Thermo Scientific). The samples were washed on a trap column (Acclaim Pepmap 100 C-18, 5 mm × 300 μm, 5 μm, 100 Å, Dionex) for 4 min with washing solution (3% (v/v) ACN, 0.1% TFA) at a flow rate of 30 μL/min prior to peptide separation using an Acclaim PepMap 100 C18 analytical column (50 cm × 75 μm, 2 μm, 100 Å, Dionex). A flow rate of 300 nL/min using eluent A (0.05% formic acid (FA)) and eluent B (80% ACN, 0.04% FA) was used for gradient separation (180 min gradient, 5–40% eluent B). Spray voltage applied on a metal-coated PicoTip emitter (10 μm tip size, New Objective, Woburn, Massachusetts, US) was 1.7 kV with a source temperature of 250 °C. Full scan MS spectra were acquired between 300 and 2000 *m*/*z* at a resolution of 70,000 at *m*/*z* 400. The 10 most intense precursors with charge states greater than 2+ were selected with an isolation window of 1.4 *m*/*z* and fragmented by HCD with normalized collision energies of 33 at a resolution of 17,500. Lock mass (445.120025) and dynamic exclusion (30 s) were enabled.

The MS raw files were processed by Proteome Discoverer 2.4 (Thermo, version 2.4.1.15), and MS/MS spectra were searched using the Sequest HT algorithm against a database containing common contaminants and the canonical mouse database. The enzyme specificity was set to semi-ArgC with two missed cleavages allowed. An MS1 tolerance of 10 ppm and a MS2 tolerance of 0.02 Da was implemented. Oxidation (15.995 Da) of methionine residues, acetylation (42.011 Da), and TMT6plex (229.163 Da) on the peptide N-terminus were set as a variable modification, while carbamidomethylation (57.02146 Da) on cysteine residues and TMT6plex on lysine residues was set as a static modification. Technical injection replicates were set as fractions. Minimal peptide length was set to 6 amino acids, and the peptide false discovery rate (FDR) was set to 1%. Normalized, scaled abundance from Proteome Discoverer was exported and log 2-transformed, and statistical analysis (*t*-test) was performed in Perseus (Perseus_1.6.10.43). To compensate for the multiple testing hypothesis, a permutation-based FDR value of 0.05 and an s0 value of 0.1 (in essence a fold change) were utilized.

### Generation of Cleavage Specificity ICElogo

The cleavage specificity logo was generated for significant cleavage events identified through N-terminomics. Each amino acid between P4 and P4′ (according to Schechter-Berger nomenclature^14^) was compared to the frequency distribution in a reference set generated from all murine proteins annotated in uniprot to calculate the percentage difference. This difference is represented by the height of the letters (using the one-letter code for amino acids with a minimum frequency of 0.06).

Color coding is as follows: red for acidic residues, blue for basic residues, black for hydrophobic residues (including alanine and proline), and green for polar residues (including tyrosine and glycine).

### Statistical Analysis and Illustrations

All statistical analyses were performed with GraphPad Prism 10 software. The particular test, which was conducted, is always stated in the respective figure descriptions (ns: *p* > 0.05; *: *p* ≤ 0.05; **: *p* ≤ 0.01; ***: *p* ≤ 0.001). The figures were created with Microsoft PowerPoint and BioRender.com.

## Results

### Identification of Novel Astrocytic Meprin β Substrates

**Figure 1:**
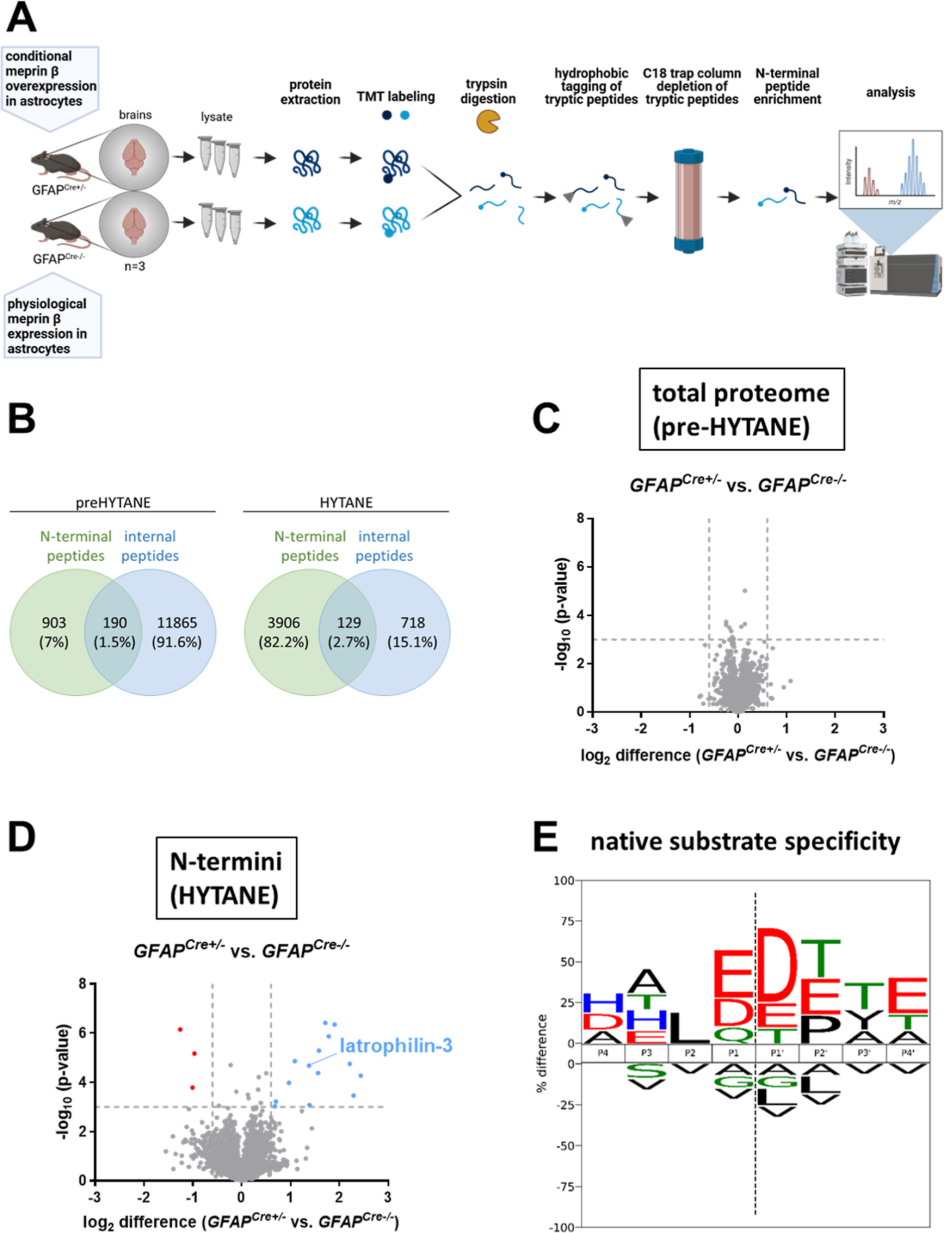
Identification of novel astrocytic meprin β substrates employing N-terminomics. (A) Cartoon of the N-terminomics workflow. (B) Quantification of identified peptides in preHYTANE and HYTANE analysis. Absolute and relative numbers of N-terminal (green) and internal (blue) peptides show the enrichment of N-terminal peptides during HYTANE. (C) Volcano plot of the preHYTANE (*n* = 3) results. The threshold was set at log_2_(difference) = ± 0.6 and −log_10_(*p*-value) = 3. All data points are below the threshold. (D) Volcano plot of the HYTANE results (*n* = 3). The threshold was set again at log_2_ (difference) = ± 0.6 and −log_10_ (*p*-value) = 3. Data points with log_2_ (difference) > 0.6 and −log_10_ (*p*-value) > 3 are colored in blue. Data points with log_2_ (difference) <−0.6 and −log_10_ (*p*-value) > 3 are colored in red. (E) Cleavage specificity logo highlights the amino acid abundance around the cleavage site, based on significantly elevated N-termini in GFAP^Cre+/–^ samples shown in (D).

In a previous study characterizing mice overexpressing meprin β in astrocytes, we conducted mouse behavior tests and observed hyperactivity and altered exploratory behavior.^5^ To analyze the underlying reason, we conducted N-terminomics by HYTANE of brain lysates from *GFAP*^*Cre*^*;Rosa26*^*Mep1b-HA*^ and respective Cre-negative control mice to identify putative meprin β substrates (Figure 1A). Employing the HYTANE strategy,^15^ 3906 new N-terminal peptides were identified, compared to 903 in the preHYTANE analysis (Figure 1B). In the preHYTANE analysis, we detected no significant differences in the protein abundance (Figure 1C). However, HYTANE analysis revealed 17 significantly altered N-termini between meprin β overexpressing and control brains [cutoff was set to −log10 (*p*-value) = 3 and log_2_ (difference) = ± 0.6] (Figure 1D). In the volcano plot the three data points corresponding to underrepresented N-terminal peptides are colored in red, whereas the 14 overrepresented peptides are colored in blue. We generated a cleavage specificity logo with the 14 overrepresented cleavage events to evaluate whether direct meprin β cleavage might have occurred (Figure 1E). Indeed, the cleavage specificity logo shows preference for acidic amino acids in P1 and P1′ sites fitting to the preference of meprin β.^16^

As we are interested in direct cleavage by meprin β, we focused on overrepresented extracellular located cleavage sites with acidic amino acids in P1 and/or P1′. These cleavage events are summarized in Table 1.

**Table 1:** Putative Meprin β Substrates Identified With N-Terminomics^a^

identified novel substrate (*gene name*)	UniProt ID, protein entry name	identified cleavage sites (P4–P4′)	described function(s)
receptor-type tyrosine-protein phosphatase ζ (*Ptprz1*)	B9EKR1 PTPRZ_MOUSE	DNEE_439_↓_440_DTGL	negatively regulates oligodendrocyte precursor proliferation in the embryonic spinal cord^17^
neurocan core protein (*Ncan*)	P55066 NCAN_MOUSE	AGDQ_24_↓_25_DTQD, QDTQ_27_↓_28_DTTA, DTQD_28_↓_29_TTAT	may modulate neuronal adhesion and neurite growth during development by binding to neural cell adhesion molecules^18^
brevican core protein (*Bcan*)	Q61361 PGCB_MOUSE	STPE_417_↓_418_DPAE, LEAL_460_↓_461_EEEK, EALE_461_↓_462_EEKE, RELE_584_↓_585_TPSE	may inhibit neurite outgrowth^19^
chondroitin sulfate proteoglycan-5 (*Cspg5*)	Q71M36 CSPG5_MOUSE	AAGE_70_↓_71_DETS	may be involved in neuritogenesis^20^
**latrophilin-3(*Adgrl3*)**	**Q80TS3 AGRL3_MOUSE**	**IHLD_484_↓_485_SELE**	**plays a role incell–celladhesion and neuron guidance^12^**
probable G-protein coupled receptor-158 (*Gpr158*)	Q8C419 MGLYR_MOUSE	GASL_26_↓_27_DPPG	orphan receptor which influences neuron morphology and controls stress-induced depression^21^
hyaluronan and proteoglycan link protein-1 (*Hapln1*)	Q9QUP5 HPLN1_MOUSE	HHLS_20_↓_21_DSYT, HLSD_21_↓_22_SYTP	part of the extracellular matrix in the brain; induces human neocortex folding^22^

a Further analyzed substrate latrophilin-3 is highlighted in bold. The order is based on the UniProt IDs, listed alphabetically.

Among all putative substrates identified by N-terminomics, latrophilin-3 was the most promising candidate to explain the behavioral phenotype of the meprin β overexpressing mice, as deficiency of latrophilin-3 in mice leads to hyperactivity^6^ and thus might be associated with the observed increased locomotion in *GFAP*^*Cre+/–*^*;Rosa26*^*Mep1b-HA*^ mice.

### Latrophilin-3 is a Newly Identified Astrocytic Substrate of Meprin β

**Figure 2:**
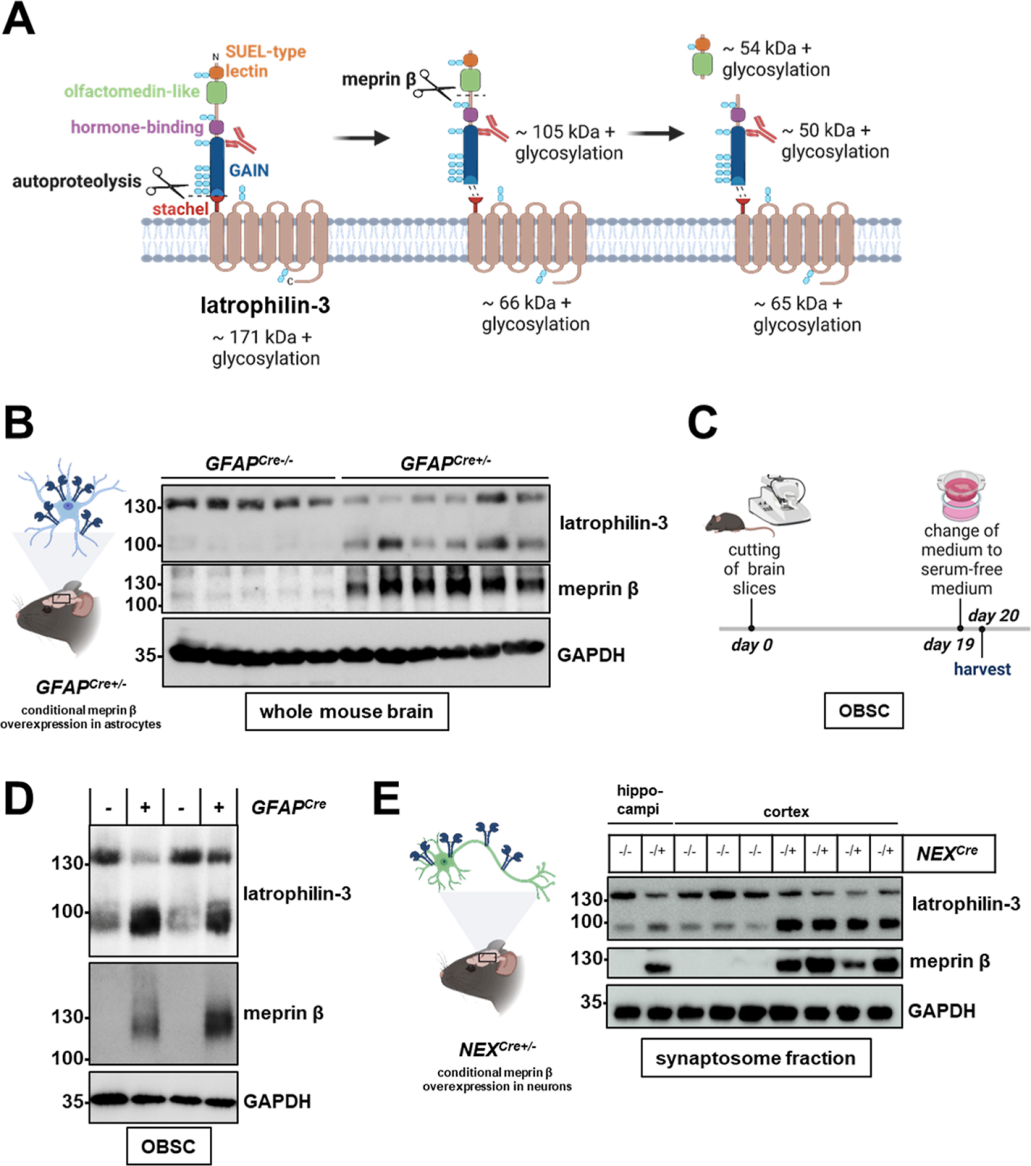
Latrophilin-3 cleavage validated in mouse brain lysates and ex vivo cultivated organotypic brain slices. (A) Latrophilin-3 consists of a short C-terminal cytosolic part, a seven-pass transmembrane domain, a GPCR autoproteolysis inducing (GAIN) domain with N-terminally adjacent hormone-binding domain, including a C-terminal stachel sequence, followed by an olfactomedin-like domain and a sea urchin egg lectin (SUEL)-type lectin domain. Upon autoproteolysis, latrophilin-3 is cleaved into a membrane-bound CTF (latrophilin-3-CTF) and a noncovalently linked N-terminal fragment (latrophilin-3-NTF). Meprin β cleaves within the latrophilin-3 ectodomain releasing a fragment containing the SUEL-type lectin and olfactomedin-like domain. The used latrophilin-3 (B-6) antibody (red) binds between the GAIN and the olfactomedin-like domain, as indicated. Glycosylation sites are highlighted in light blue. The molecular weight of latrophilin-3 and its cleavage fragments are depicted based on the amino acid sequence; however, the actual molecular mass is presumably higher than calculated due to glycosylated side chains. (B) Brains from one-year-old *GFAP*^*Cre*^*;Rosa26*^*Mep1b-HA*^ mice (*GFAP*^*Cre+/–*^) with a conditional overexpression of meprin β in astrocytes and control mice (*GFAP*^*Cre–/–*^) were homogenized. Lysates were analyzed with SDS-PAGE and Western blot. (C) Generation and cultivation procedure of organotypic brain slices. Mice were sacrificed and the brain was isolated and cut in 250 μm thick slices with a vibratome. Then the organotypic brain slices were cultivated for 19 days in serum-supplemented medium. As serum components inhibit meprin β activity, the medium was substituted by serum-free media for 24 h prior harvesting. (D) Brains from *GFAP*^*Cre*^*;Rosa26*^*Mep1b-HA*^ mice (NEX^Cre+/–^) and control mice (were cut into 250 μm sections. The OBSCs were cultivated for 20 days and lysates were analyzed by SDS-PAGE and Western blot. (E) Synaptosome fractions of hippocampi or cortex of *NEX*^*Cre*^*;Rosa26*^*Mep1b-HA*^ mice (*NEX*^*Cre+/–*^), showing a conditional overexpression of meprin β in neurons and control mice (*NEX*^*Cre–/–*^) were prepared and analyzed with SDS-PAGE and Western blot.

Recent studies showed that the GPCR latrophilin-3 is involved in neuronal guidance, which is important for the formation of synapses.^12^ It undergoes autoproteolysis in the extracellular domain proximal to the cell membrane.^23−25^ However, both resulting latrophilin-3 fragments remain associated by noncovalent interactions (Figure 2A). According to the N-terminomics data, meprin β cleaves latrophilin-3 between its olfactomedin-like and hormone-binding domains between D484 and S485. Indeed, a matching membrane-attached cleavage fragment of around 90 kDa appeared in meprin β overexpressing mouse brains (Figure 2B) and was increased in respective OBSCs (Figure 2C and D), validating latrophilin-3 as a substrate of meprin β in astrocytes. However, latrophilin-3 is expressed not only in glia cells but also in neurons.^26^ To check for a cleavage of latrophilin-3 by meprin β in neurons, we additionally analyzed mice overexpressing meprin β in neurons of the hippocampus and cortex (*NEX*^*Cre+/–*^) (Figure 2E). Comparing synaptosome fractions of hippocampi and cortex of *NEX*^*Cre+/–*^ to the respective controls (*NEX*^*Cre–/–*^), we could detect an increase in a cleavage fragment at the same molecular weight as observed for the meprin β overexpression in astrocytes (at around 90 kDa). Thus, latrophilin-3 is obviously not exclusively shed by meprin β in astrocytes but also within other cell types.

### Validation of Latrophilin-3 Cleavage by Membrane Bound Meprin β in Cellulo

**Figure 3:**
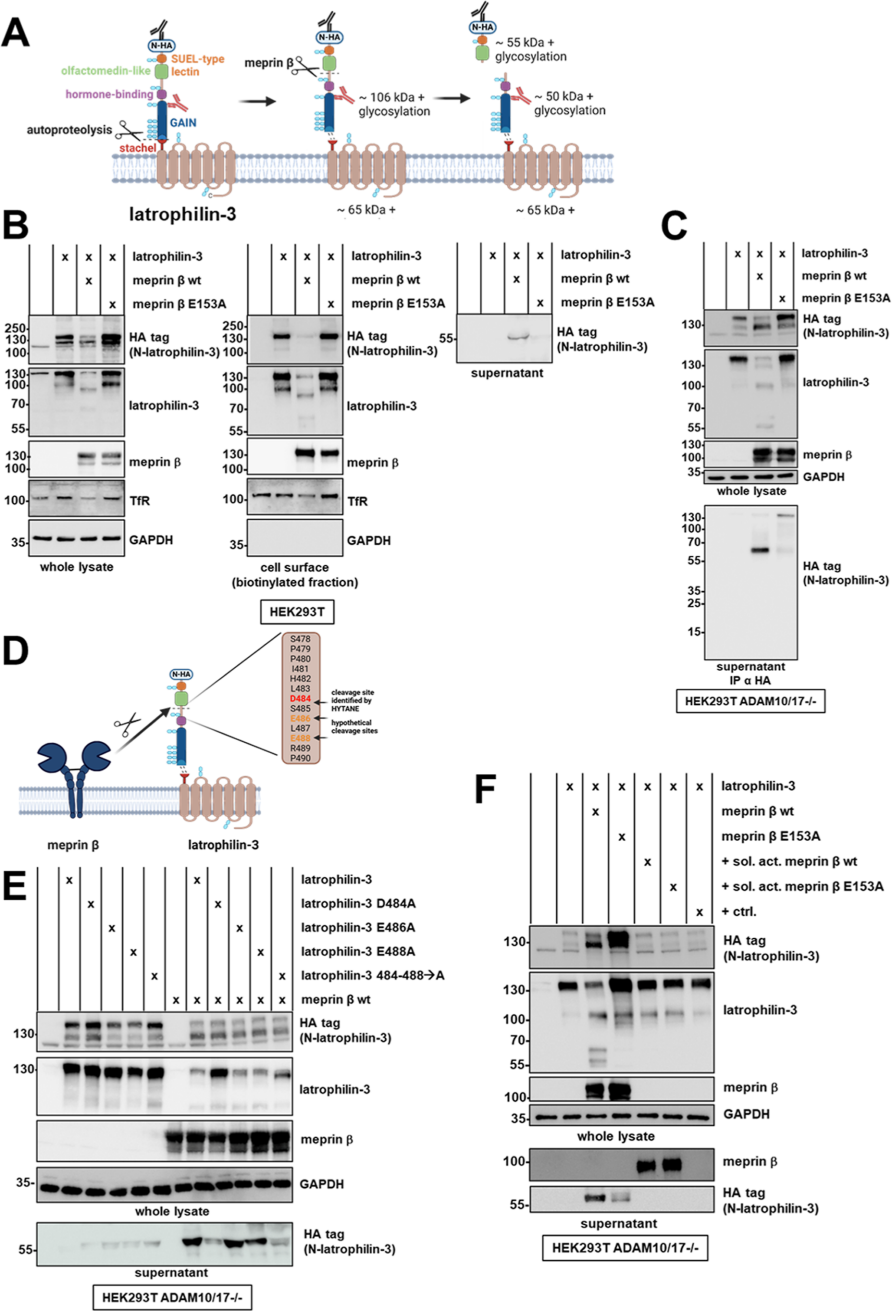
Latrophilin-3 cleavage validated in transfected HEK cells. (A) Latrophilin-3 consists of a short C-terminal cytosolic part, a seven-pass transmembrane domain, a GPCR autoproteolysis inducing (GAIN) domain with N-terminally adjacent hormone-binding domain, including a C-terminal stachel sequence, followed by an olfactomedin-like domain and a sea urchin egg lectin (SUEL)-type lectin domain. The latrophilin-3 expressed from the used plasmid is N-terminally HA tagged (N-HA). Upon autoproteolysis, latrohilin-3 is cleaved into a membrane-bound CTF (Latrophilin-3-CTF) and a noncovalently linked N-terminal fragment (Latrophilin-3-NTF). Meprin β cleaves within the latrophilin-3 ectodomain releasing a fragment containing the SUEL-type lectin and olfactomedin-like domain. The used latrophilin-3 (B-6) antibody (red) binds between the GAIN and the olfactomedin-like domain as indicated. The N-terminal HA tag can be detected with a HA antibody (black). Glycosylation sites are highlighted in light blue. The molecular weight of latrophilin-3 and its cleavage fragments are depicted based on the amino acid sequence; however, the actual molecular mass is presumably higher than calculated due to glycosylated side chains. (B) HEK cells were transfected with human wt meprin β, inactive meprin β E153A and latrophilin-3. After 24 h, the medium was changed to serum-free DMEM for 4 h. Cells were biotinylated, harvested, and lysed. The whole lysate, the biotinylated fraction, as well as supernatants were analyzed by SDS-PAGE and Western blot. (C) HEK cells deficient for ADAM10 and ADAM17 were transfected with human wt meprin β, inactive meprin β E153A, and latrophilin-3. After 24 h, the medium was changed to serum-free DMEM for 24 h. Cells were lysed, and cleavage fragments of latrophilin-3 were immunoprecipitated using a HA antibody. The whole lysates as well the immunoprecipitated proteins were analyzed by SDS-PAGE and Western blot. (D) Cleavage site of meprin β within latrophilin-3 identified by HYTANE and hypothetical cleavage sites in close proximity matching the cleavage specificity of meprin β. (E) Different variants of latrophilin-3 harboring amino acids exchanges within the region of cleavage by meprin β were generated by site-directed mutagenesis. HEK cells deficient for ADAM10 and ADAM17 were transfected with latrophilin-3 variants alone or together with wt meprin β. After 24 h, the medium was changed to serum-free DMEM for 24 h. Cells were lysed, and whole lysates as well the supernatants were analyzed by SDS-PAGE and Western blot. (F) HEK cells deficient for ADAM10 and ADAM17 were transfected with human wt meprin β and inactive meprin β E153A, together with latrophilin-3. After 24 h, the medium was changed to serum-free DMEM for 24 h. Cells only transfected with latrophilin-3 were treated with purified, soluble activated wt meprin β or inactive meprin β E153A or a control (trypsin/ovomucoid) for 24 h after medium was changed to serum-free DMEM. Cells were lysed, and the whole lysates as well the immunoprecipitated proteins were analyzed by SDS-PAGE and Western blot.

Further, we used HEK293T cells for transient transfection to validate proteolysis of latrophilin-3 by meprin β. For this purpose, N-terminally HA tagged latrophilin-3 (Figure 3A) was cotransfected with either wt meprin β or a catalytically inactive variant (meprin β E153A) as control, clearly demonstrating direct cleavage of latrophilin-3 by wt meprin β. A cell surface biotinylation experiment revealed that full-length latrophilin-3 located at the plasma membrane was decreased upon coexpression with wt meprin β using both the HA tag and latrophilin-3 antibody (Figure 3B). Moreover, latrophilin-3 cleavage fragments of 90 kDa and around 60 kDa were detected in the biotinylated fraction. At the same time, an N-terminal cleavage fragment of latrophilin-3 of around 55 kDa was released into the supernatant, upon coexpression with wt meprin β. The catalytically inactive meprin β variant E153A did not lead to a decrease of full-length latrophilin-3 nor a release of a latrophilin-3 cleavage fragment into the supernatant. In the absence of wt meprin β, an additional latrophilin-3 signal appeared at 100 kDa. As this signal was not detected via the N-terminal HA tag, it presumably represents a N-terminally cleaved latrophilin-3 fragment that is independent of meprin β suggesting that also other proteases may be capable of cleaving latrophilin-3. Therefore, we additionally cotransfected other proteases, namely, meprin α, ADAM10, ADAM17, BACE1, and MT1-MMP together with latrophilin-3 (Supporting Information Figure S1). This overexpression experiment showed that also meprin α, ADAM10, and MT1-MMP may proteolytically process latrophilin-3. Thus, to further study the cleavage of latrophilin-3 by meprin β in more detail, we used HEK293T cells deficient for ADAM10 and ADAM17 to avoid an effect of endogenously expressed ADAM10/17. To further investigate cleavage site(s) and cleavage fragments(s) generated by meprin β, we additionally performed an enrichment of N-terminal fragments from the supernatant of cells transfected with latrophilin-3 and meprin β using immunoprecipitation with an anti HA tag antibody and magnetic beads (Figure 3C). However, also in this experiment, only one particular signal at around 55 kDa could be detected, supporting that meprin β is only able to cleave latrophilin-3 in the confined area between the hormone binding domain and the olfactomedin-like domain.

Our N-terminomic approach only detected significant differences between meprin β overexpressing mice and control mice for one neo N-terminus of latrophilin-3, suggesting that cleavage only takes place between D484 and S485 (Table 1, P4–P4′: IHLD_484_↓_485_SELE). This cleavage site nicely fits to the preference of meprin β for acid amino acids in P1 and P1′ position. However, as latrophilin-3 harbors additional acidic aspartate residues at positions 486 and 488, we hypothesized that meprin β might also cleave latrophilin-3 at these sites further C-terminally of the site identified in the N-terminomic approach. Therefore, we generated variants of latrophilin-3 with mutations at the cleavage site identified by HYTANE (D484A) and the additionally hypothesized cleavage sites E486A and E488A by site-directed mutagenesis (Figure 3D). As alanine is disfavored in P1 and P1′ positions by meprin β, we would expect a decreased shedding when acidic amino acids at the cleavage sites are replaced by alanine residues. Additionally, we generated a variant where amino acids 484 to 488, the whole region where we could expect cleavage to take place, are replaced by alanine residues. Co-transfection of these variants with meprin β showed that amino acid exchanges E486A and E488A did not influence shedding of latrophilin-3 (Figure 3E). However, the D484A as well as the variant with amino acids 484 to 488 mutated to alanine residues show a nearly complete abolished cleavage by meprin β. This nicely validates the cleavage of latrophilin-3 by meprin β exclusively between D484 and S485.

Meprin β is primarily present at the plasma membrane in its membrane bound form but can also be shed in its pro-form by ADAM10, ADAM17, and MT1-MMP.^27,28^ Notably, membrane-bound and soluble meprin β have access to different substrate pools.^2,29^ Thus, it was of interest to analyze if also soluble meprin β is able to proteolytically process latrophilin-3 (Figure 3F). Therefore, HEK cells deficient for ADAM10 and ADAM17 were either cotransfected with latrophilin-3 and meprin β, a condition where meprin β is only present in its membrane bound form, or transfected with latrophilin-3 alone and treated with purified soluble active meprin β. For soluble meprin β-treated samples, no latrophilin-3 cleavage fragments could be detected. Thus, only membrane-bound and not the soluble form of meprin β seems to be able to process latrophilin-3.

## Discussion

Meprin β is a metalloprotease upregulated in brains of AD patients, causing neurotoxic Aβ generation through cleavage of APP at the β-secretase site.^2,3^ Knocking out meprin β in an AD mouse model results in reduced Aβ formation, decreased plaque burden, and recovered memory deficits.^3^

Recently, we published a mouse model that overexpresses meprin β in astrocytes to mimic the elevated meprin β levels observed in AD.^5^ This model exhibited an increased Aβ production and mild cognitive impairments. However, plaque deposition and severe memory deficits were not observed. Interestingly, we detected increased locomotion in various behavioral tests and altered exploratory behavior in an Open field test, which cannot necessarily be attributed to APP cleavage. Thus, we speculate also an APP-independent role for meprin β in brain function. Interestingly, previous research has also linked meprin β to cerebral function, as the MEP1B gene is associated with some severe cognitive disabilities. Specifically, the T324A mutation in meprin β, identified in diagnostic exome sequencing, has been correlated with low IQ.^30^ To further investigate the role of meprin β in the brain, particularly in astrocytes, we conducted N-terminomics of brain lysates of *GFAP*^*Cre+/–*^*;Rosa26*^*Mep1b-HA*^, and the respective Cre-negative mice to identify substrates that might cause or contribute to the observed behavior changes. Using HYTANE, we enriched 3906 N-termini from 903 N-termini in preHYTANE. From these peptides, we identified seven potential new substrates of meprin β involved in brain development or neuronal function. Knockout mice for several of these proteins exhibit behavioral deficits. For instance, hyaluronan and proteoglycan link protein-1-deficient mice show impaired righting response,^31^*Cspg5*-knockout mice exhibit diminished maternal care behavior,^32^ and the absence of receptor-type tyrosine-protein phosphatase ζ leads to mild increased activity in an Open Field Test.^33^ However, among the identified substrates, latrophilin-3 might have the greatest impact on the phenotype of the mice overexpressing meprin β in astrocytes, as it was associated with ADHD in several genetic studies.^7−9^ Consistent with these findings, *Adgrl3* knockout mice exhibit altered neurotransmitter levels and hyperactivity.^6^

Thus, in our meprin β-overexpressing mice, this effect might be caused by meprin β-mediated latrophilin-3 cleavage. The consequences of the observed cleavage event for the latrophilin-3 function are not yet known. Since the brain variant of latrophilin-3 is not capable of inducing signaling,^10^ this cannot be the underlying reason for the behavioral phenotype. Instead, it has been shown that latrophilin-3 on radial glia cells interacts with teneurins and FLRT proteins on neurons to facilitate axonal guidance for proper synapse formation.^12^ Interestingly, these interactions are mediated by the two N-terminal SUEL-type lectin and olfactomedin-like domains. Teneurins interact with both N-terminal domains, whereas FLRT proteins only interacts with the olfactomedin-like domain of latrophilins.^12,34−36^ According to the identified cleavage site, both of these domains are cleaved off by meprin β, which we confirmed via Western blot analysis conducted in both cellular and in vivo contexts. Using site-directed mutagenesis, we were able to validate that latrophilin-3 is exclusively cleaved by meprin β between D484 and S485, the cleavage site detected by HYTANE. In a mouse model overexpressing meprin β in neurons of the hippocampus and cortex, we also observed increased proteolytic processing of latrophilin-3. Thus, not only meprin β expressed on astrocytes but also on neurons can cleave latrophilin-3. Since meprin β also exists as a soluble protein, it is possible that the protease cleaves latrophilin-3 even on cells that do not express meprin β. However, as we were able to show that soluble meprin β is not able to proteolytically process latrophilin-3, meprin β shed from the cell surface of, for example, astrocytes, cannot be responsible for the cleavage of latrophilin-3 on other cell types. At least from our in cellulo experiments, other proteases, like meprin α, ADAM10, and MT1-MMP can proteolytically process latrophilin-3. Notably, these proteases are known to interact within a complex protease web, influencing each other in their shedding, activity and the processing of proteolytic substrates.^27,28,37^ Thus, further studies are needed to elucidate latrophilin-3 shedding within this well-orchestrated proteolytic web in more detail under distinct conditions in health and disease, for example, regarding compensation or regulatory effects. Additionally, it would be interesting to further investigate effects of known SNVs within these protease genes.^38^ The identified T324A variant in meprin β, which was correlated with low IQ,^30^ however, showed no differences regarding the processing of latrophilin-3 compared to wt meprin β. Thus, the possible detrimental effect of this variant is probably due to the altered cleavage of other substrates or interaction partners of meprin β or may only become apparent on a longer time scale in vivo.

Interestingly, not only mice overexpressing meprin β in astrocytes but also meprin β-deficient mice show increased hyperactivity.^31^ The reason for the seemingly contradictory effects of dysregulated protease activity requires further investigation and probably cannot be attributed only to the interaction of meprin β and latrophilin-3. Most likely, the altered processing of other, yet unknown, substrates also may play a role. Additionally, meprin β interacts with other proteases, inhibitors, and regulatory factors within a complex protease web.^39^ Proteases can regulate each other through limited proteolysis, so the absence of one enzyme can alter the function of others, as known for the interaction of meprins and ADAMs.^28,37^ On the contrary, in the overexpression models, increased protease activity may lead to enhanced cleavage of other proteases, probably also partially inactivating them and disrupting proteolytic balance as well. Furthermore, in knockout models, compensatory upregulation of other proteases may occur, thereby influencing overall proteolytic activity. To further address increased hyperactivity in mice lacking or overexpressing meprin β also, the effects on inhibitors need to be studied. For example, high levels of a protease may trigger an increased expression of endogenous inhibitors, ultimately blocking its function. Since many processes rely on transient proteolysis followed by inhibition of protease activity,^40^ both knockout and excessive protease activity may result in similar phenotypes. This observation of the hyperactivity promoting effect of both overexpression and knockout of meprin β also limits the usability of meprin β as a therapeutic target in this case.

In summary, we could show that in vivo within the brain meprin β cleaves latrophilin-3, leading to the loss of the interaction domains of latrophilin-3. Consequently, inadequate synapse formation may result, potentially contributing to the hyperactivity observed in the behavior tests. Further elucidation of the detailed mechanisms underlying this process will be the focus of future studies.

## Data Availability

All data needed to evaluate the conclusions in the paper are present in the paper and/or the Supporting Information. The complete data sets used and/or analyzed during the current study are available from the corresponding author on reasonable request. The mass spectrometry proteomics data have been deposited to the ProteomeXchange Consortium (http://proteomecentral.proteomexchange.org) via the PRIDE partner repository^41^ with the data set identifier PXD056646.

## Supporting Information Available

Latrophilin-3 can also be cleaved by other proteases apart from meprin β and meprin β T324A variant does not influence latrophilin-3 cleavage compared to meprin β wildtype Western blots (PDF)

## Notes

All authors consented to publish the manuscript.

## Notes

Ethics approval: All animal studies were conducted in compliance with European and German guidelines for the care and use of laboratory animals. GFAP^Cre+/–^;Rosa26^*Mep1b*–HA^ and GFAP^Cre–/–^;Rosa26^*Mep1b*–HA^ animals were kept to remove organs, approved by the Central Animal Facility of the University of Kiel and documented under number 750 in the local animal facility. This is not an animal experiment in the sense of the German Animal Welfare Act, as no animal was stressed or manipulated. Therefore, no further approval from the responsible authority (Ministry of Agriculture, Rural Areas, Europe and Consumer Protection including the Ethics Committee according to Section 15 of the Animal Welfare Act) was required. NEX^Cre+/–^;Rosa26^Mep1b–HA^ and NEX^Cre–/–;^Rosa26^Mep1b–HA^ were kept in the animal facility of the Johannes-Gutenberg University Mainz with approval by the Landesuntersuchungsamt (LUA) Rheinland-Pfalz documented under the approval number G20-1-026.

## List of Abbreviations

Aβ: amyloid-β
ACN: acetonitrile
AD: Alzheimer’s disease
ADHD: attention-deficit/hyperactivity disorder
APP: amyloid precursor protein
BACE1: β-site of APP cleaving enzyme 1
cAMP: cyclic adenosine monophosphate
DMEM: Dulbecco’s Modified Eagle Medium
DTT: dithiothreitol
EDTA: ethylenediaminetetraacetic acid
EGTA: ethylene glycol-bis(β-aminoethyl ether)-*N*,*N*,*N*′,*N*′-tetraacetic acid
FA: formic acid
FBS: fetal bovine serum
FDR: false discovery rate
FLRT: fibronectin leucine-rich transmembrane
GAPDH: glyceraldehyde 3-phosphate dehydrogenase
GFAP: glial fibrally acidic protein
GPCR: G protein-coupled receptor
HBSS: Hanks’ Balanced salt solution
HYTANE: hydrophobic tagging-assisted N-termini enrichment
IVC: individually ventilated cage
MAM: meprin/A5 protein/receptor protein tyrosine phosphatase μ
MS: mass spectrometry
OBSC: organotypic brain slice culture
PBS: phosphate-buffered saline
PEI: polyethylenimine
PFA: paraformaldehyde
SDS: sodium dodecyl sulfate
SDS-PAGE: SDS-polyacrylamide gel electrophoresis
SNP: single nucleotide polymorphism
SUEL: sea urchin egg lectin
TBS: tris-buffered saline
TCEP: tris(2-carboxyethyl)phosphin
TFA: trifluoroacetic acid
TfR: transferrin receptor
TMB: tetramethylbenzidin
TMT: tandem mass tag
wt: wildtype
