# Supplementary material for: HYTANE-Identified Latrophilin-3 Cleavage by Meprin β Leads to Loss of the Interaction Domains
Source: J Proteome Res. 2025 Mar 26;24(4):1832–44. doi: 10.1021/acs.jproteome.4c00912 (PMC11976865; doi:10.1021/acs.jproteome.4c00912)
Supplement: Supplementary file 1 — pr4c00912_si_001.pdf [file pr4c00912_si_001.pdf]

Supporting Information for

HYTANE-identified latrophilin-3 cleavage by meprin β leads to loss of the interaction domains

Fred Armbrust[1#], Kira Bickenbach[1#], Tomas Koudelka[2], Corentin Joos[1], Maximilian Keller[3], Andreas Tholey[2], Claus U. Pietrzik[3] and Christoph Becker-Pauly[1*]

[1]Biochemical Institute, Unit for Degradomics of the Protease Web, University of Kiel, 24118 Kiel, Germany,
[2]Systematic Proteomics & Bioanalytics, Institute for Experimental Medicine, University of Kiel, 24105 Kiel, Germany
[3]Institute for Pathobiochemistry, University Medical Center of the Johannes Gutenberg University Mainz, 55128 Mainz, Germany

# Both authors contributed equally to this work.

[*] Corresponding author:

Prof. Dr. Christoph Becker-Pauly, Biochemical Institute, Unit for Degradomics of the Protease Web, University of Kiel, Otto-Hahn-Platz 9, 24118 Kiel, Germany, Tel: 0049-431-880 7118, Fax: 0049-431-880 2238, E-mail: cbeckerpauly@biochem.uni-kiel.de

Table of Contents Supporting Information:

Figure S1 - Latrophilin-3 can also be cleaved by other proteases apart from meprin β

Figure S2 - Meprin β T324A variant does not influence latrophilin-3 cleavage compared to meprin β wildtype

Western blots

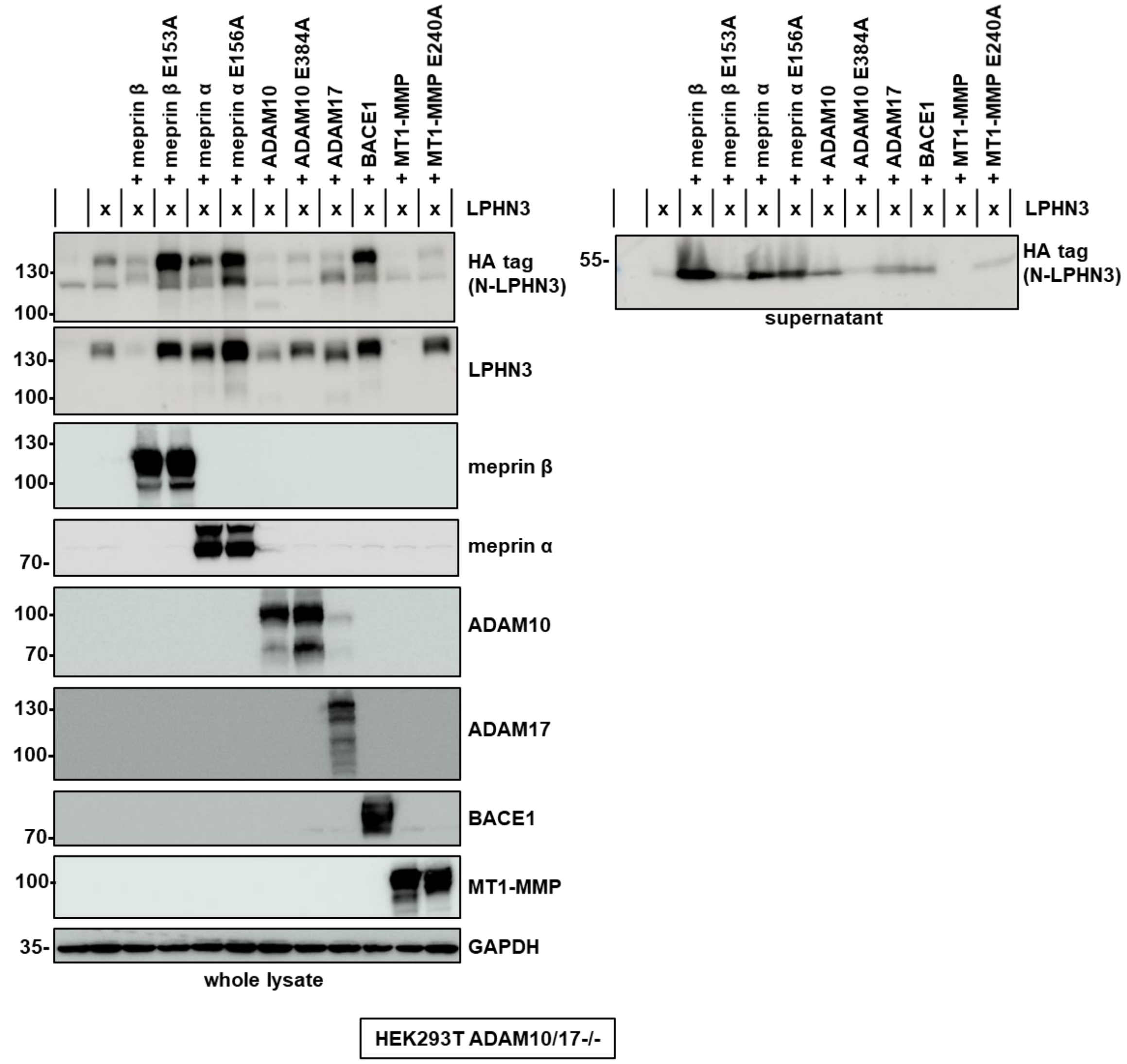

Supplementary Fig. S1: Latrophilin-3 can also be cleaved by other proteases apart from meprin β HEK cells deficient for ADAM10 and ADAM17 were transfected with human wt meprin β, inactive meprin β E153A, meprin α, inactive meprin α E156A, ADAM10, inactive ADAM10 E384A, ADAM17, BACE1, MT1-MMP, inactive MT1-MMP E240A and latrophilin-3. After 24 h, the medium was changed to serum-free DMEM for 24 h. Cells were lysed and the whole lysates, as well as supernatants were analyzed by SDS-PAGE and western blot.

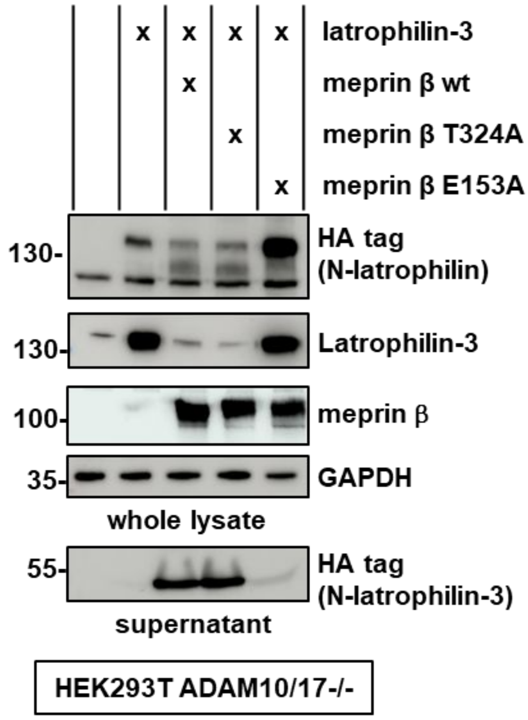

Supplementary Fig. S2: Meprin β T324A variant does not influence latrophilin-3 cleavage compared to meprin β wildtype HEK cells deficient for ADAM10 and ADAM17 were transfected with human wt meprin β, meprin β T423A, inactive meprin β E153A and latrophilin-3. After 24 h, the medium was changed to serum-free DMEM for 24 h. Cells were lysed and the whole lysates, as well as supernatants were analyzed by SDS-PAGE and western blot.

Western Blots

Original full blots of figure 2B

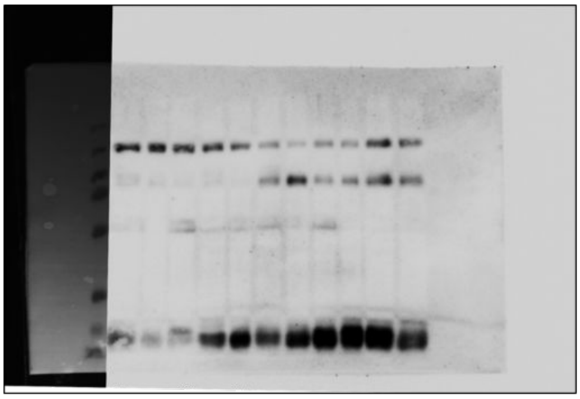

latrophilin-3

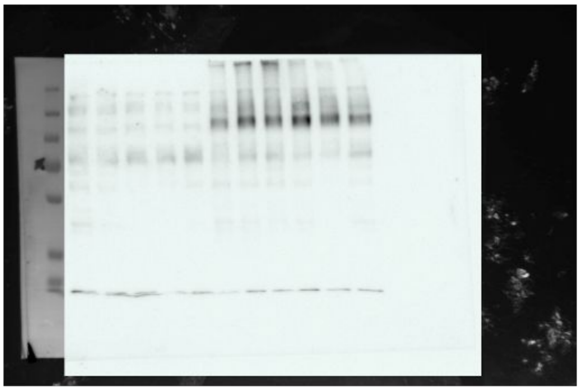

meprin β

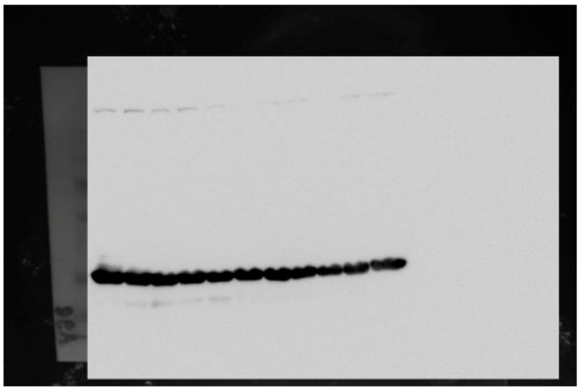

GAPDH

Original full blots of figure 2D

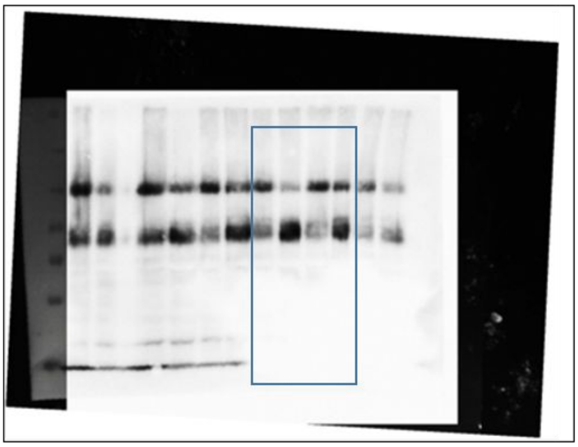

latrophilin-3

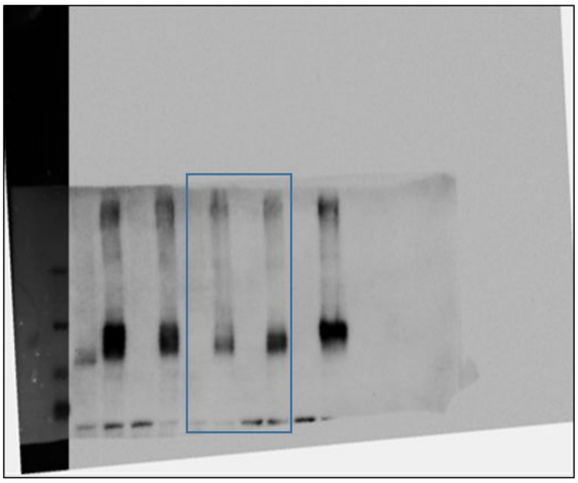

meprin β

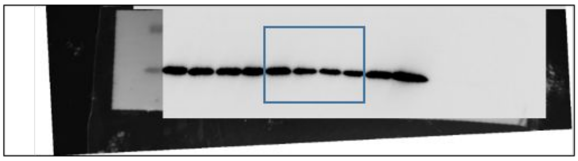

GAPDH

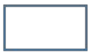 **representative section shown in manuscript**

Original full blots of figure 2E

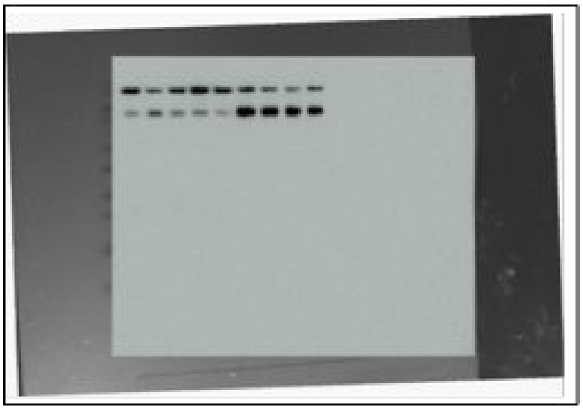

latrophilin-3

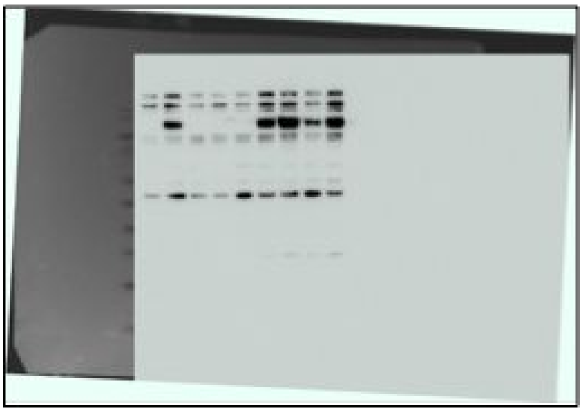

meprin β

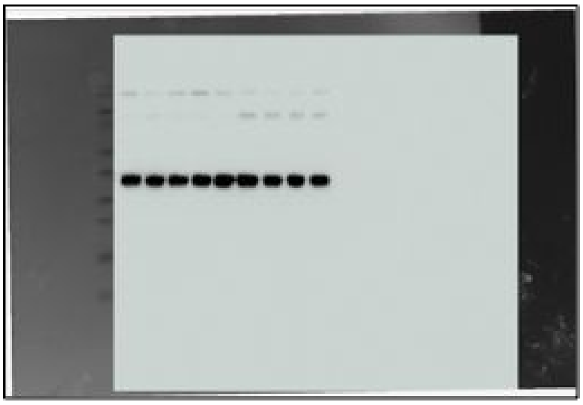

GAPDH

Original full blots of figure 3B

whole lysate:

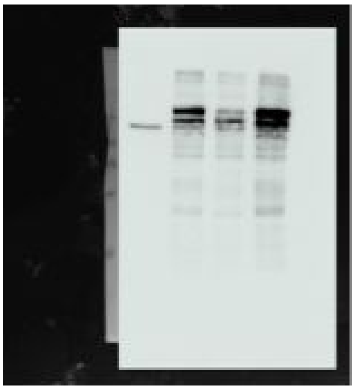

HA-tag (N-latrophilin-3)

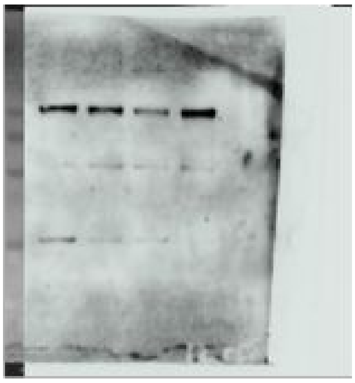

TfR

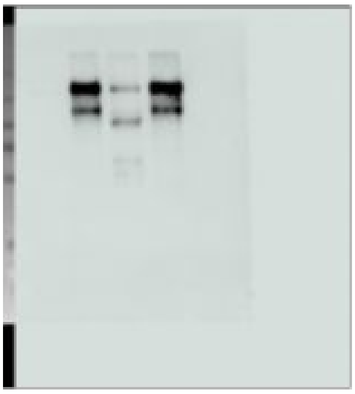

latrophilin-3

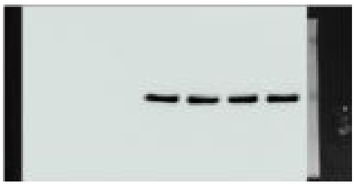

GAPDH

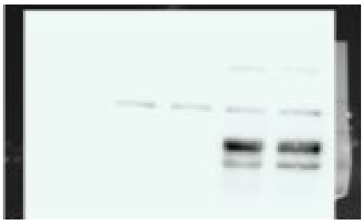

meprin β

Original full blots of figure 3B

Cell surface:

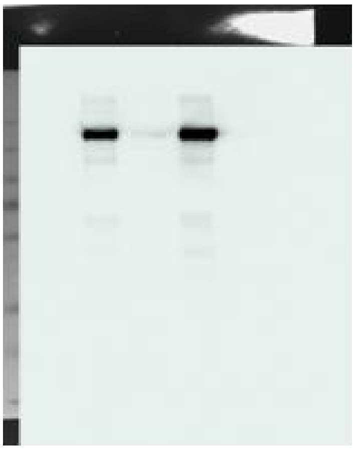

HA-tag (N-latrophilin-3)

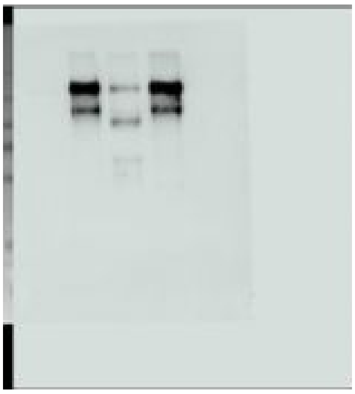

latrophilin-3

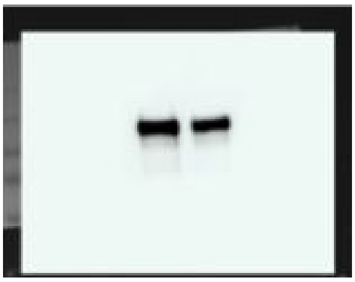

meprin β

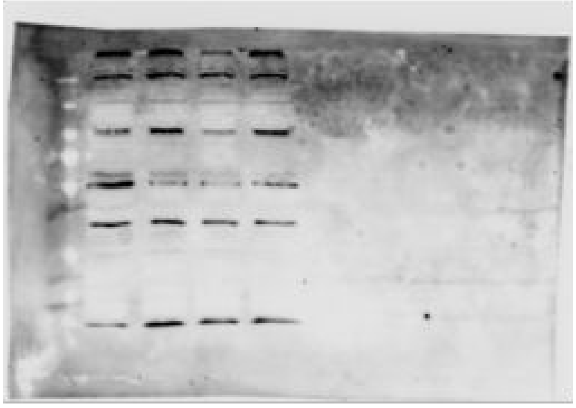

TfR

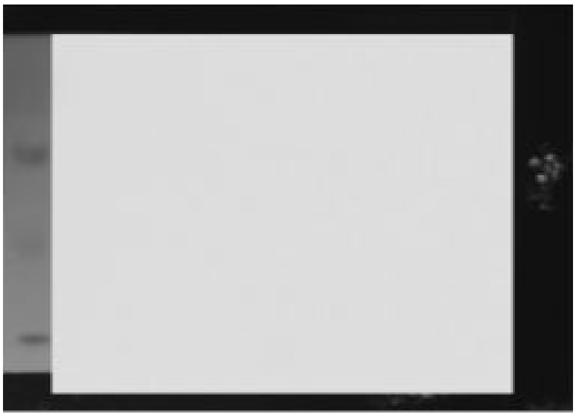

GAPDH

supernatant:

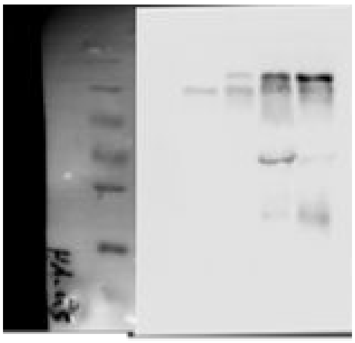

HA-tag (N-latrophilin-3)

Original full blots of figure 3C

whole lysate:

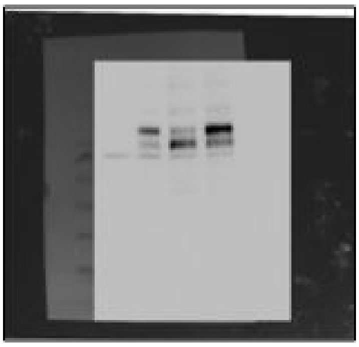

HA-tag
(N-latrophilin-3)

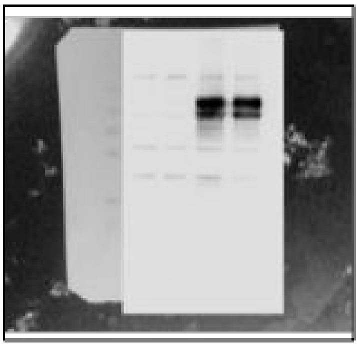

meprin β

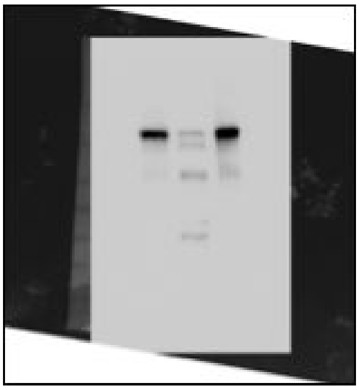

latrophilin-3

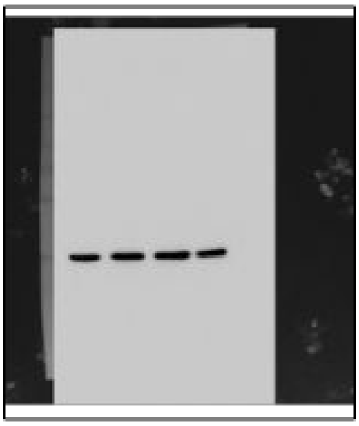

GAPDH

Supernatant IP

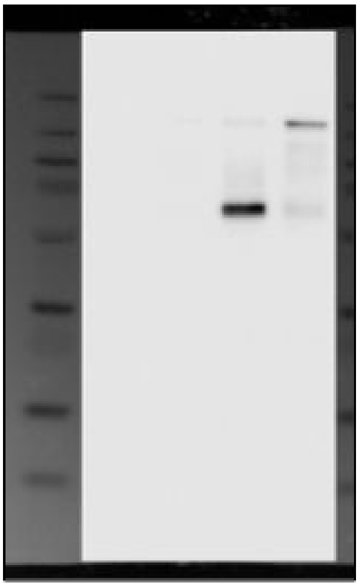

HA-tag
(N-latrophilin-3)

Original full blots of figure 3E

whole lysate

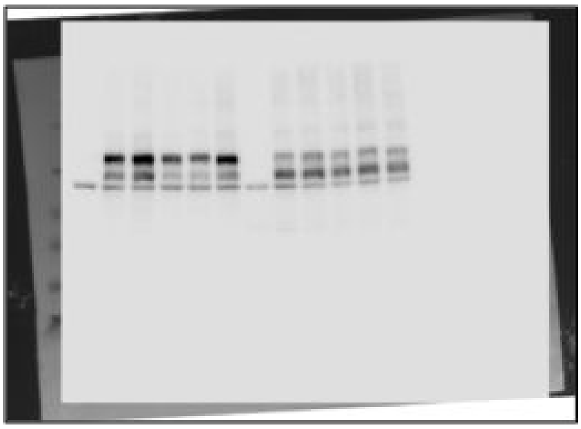

HA tag
(N-LPHN3)

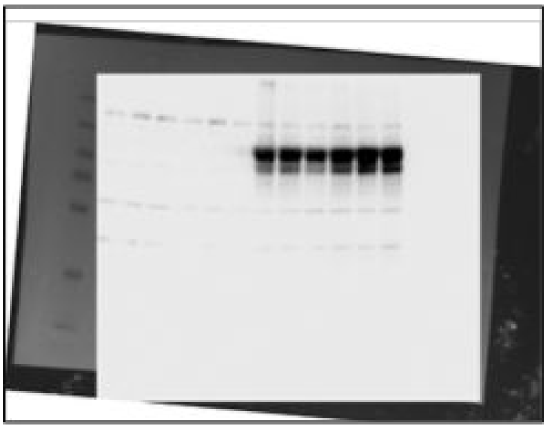

meprin β

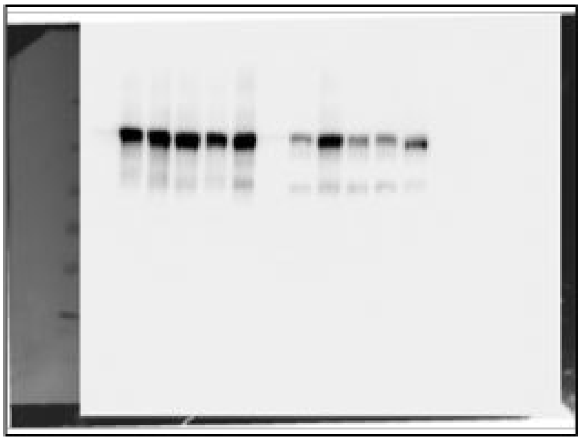

LPHN3

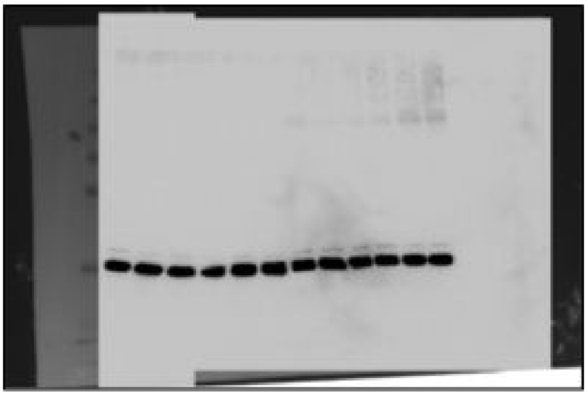

GAPDH

supernatant

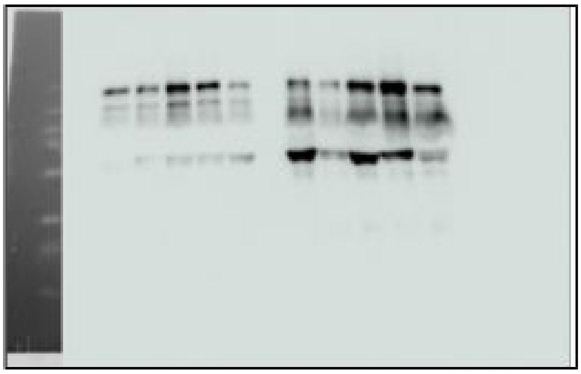

HA tag
(N-LPHN3)

Original full blots of figure 3F

whole lysate

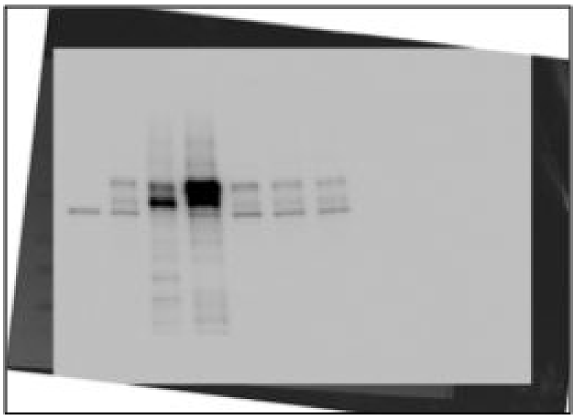

HA tag (N-LPHN3)

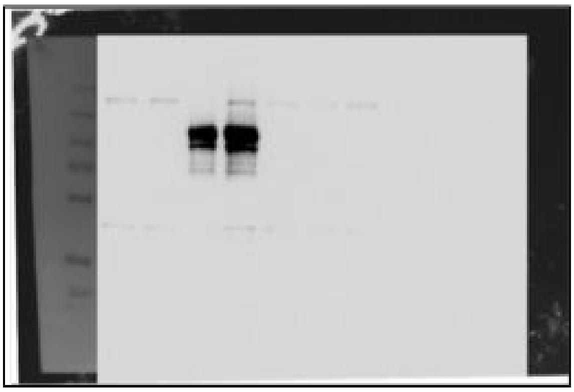

meprin β

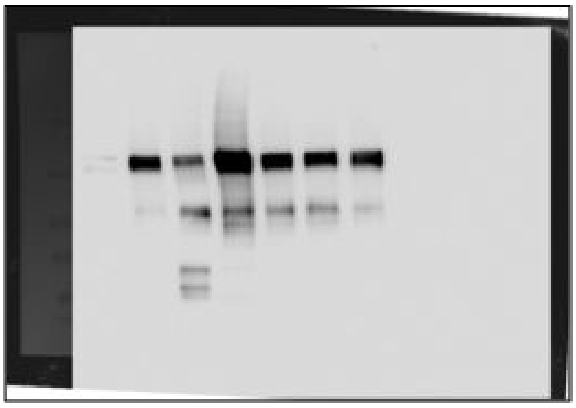

LPHN3

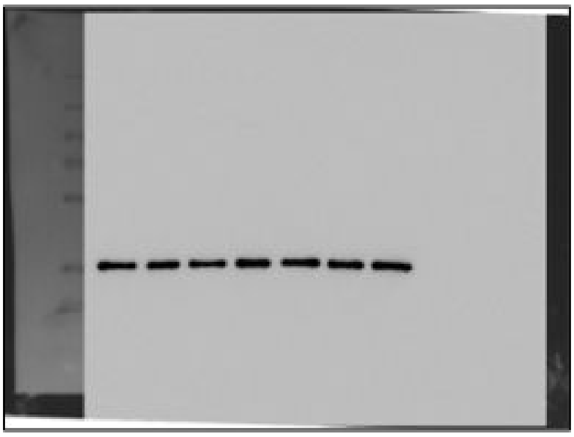

GAPDH

supernatant

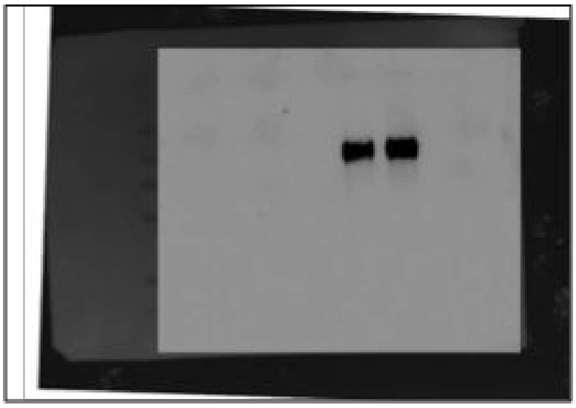

meprin β

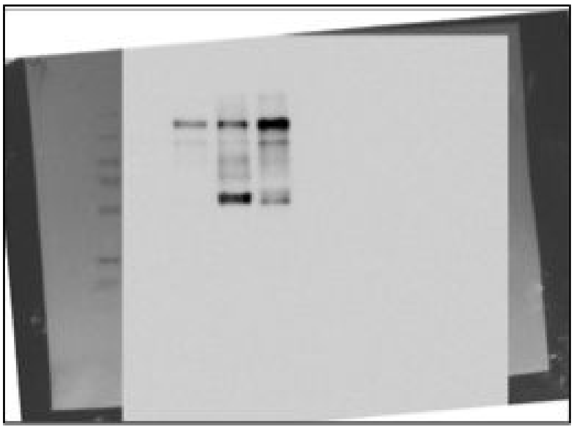

HA tag (N-LPHN3)

Original full blots of supplementary figure S1

whole lysate

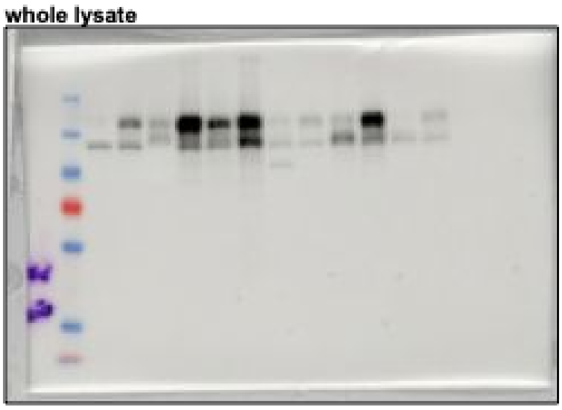

HA tag
(N-LPHN3)

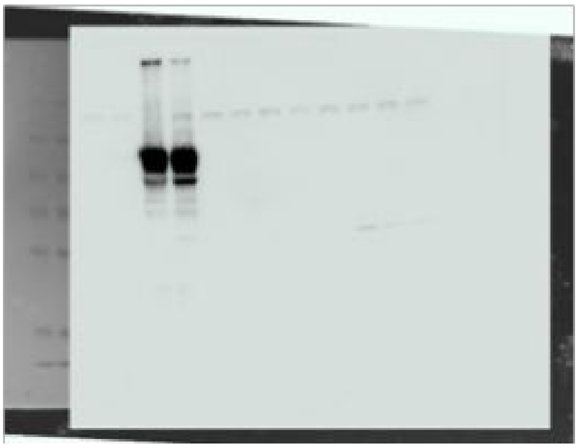

meprin β

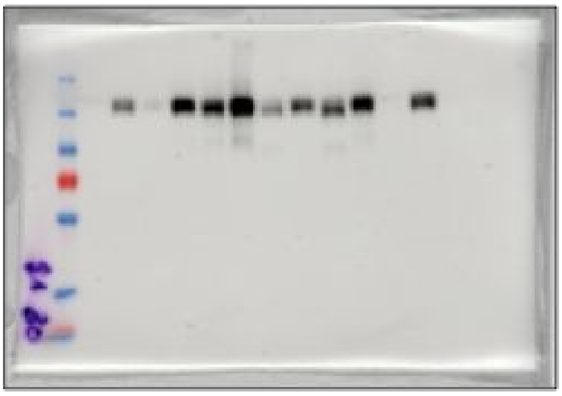

LPHN3

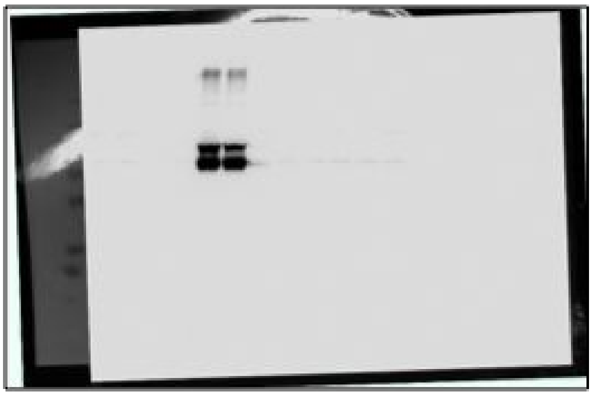

meprin α

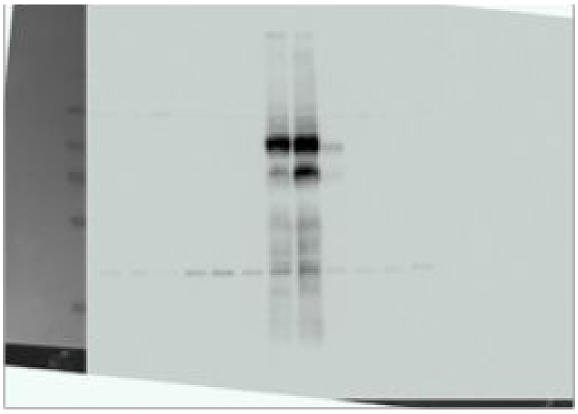

ADAM10

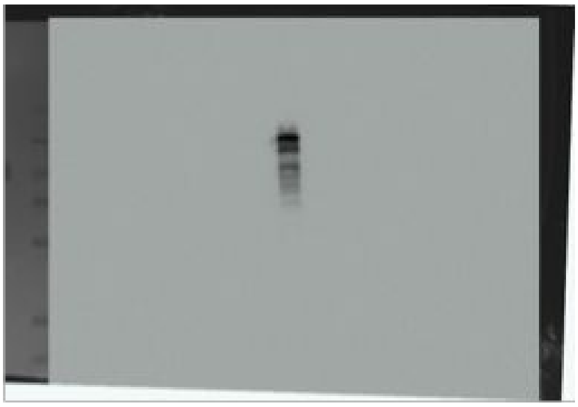

ADAM17

Original full blots of supplementary figure S1

whole lysate

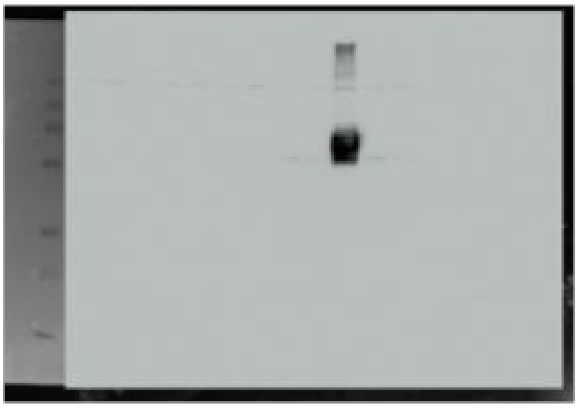

BACE1

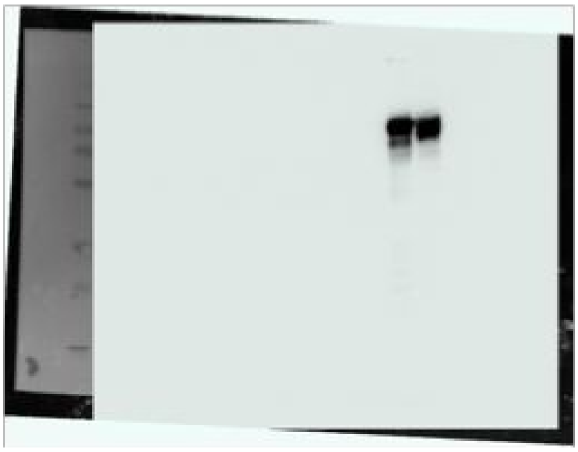

MT1-MMP

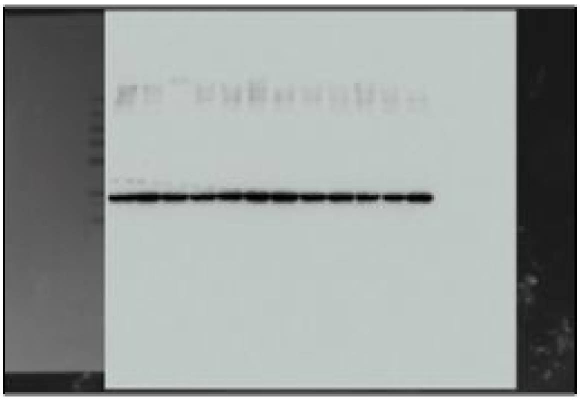

GAPDH)

supernatant

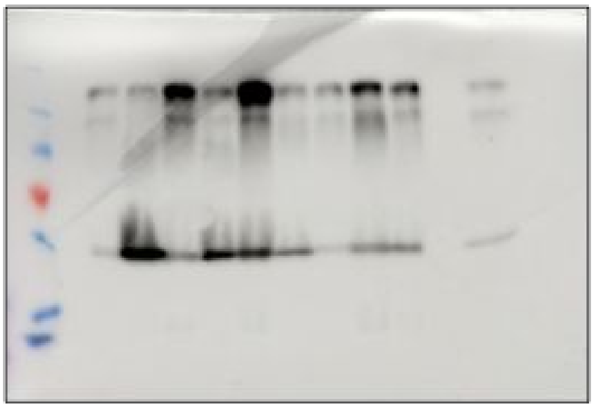

HA tag
(N-LPHN3)

Original full blots of supplementary figure S2

whole lysate

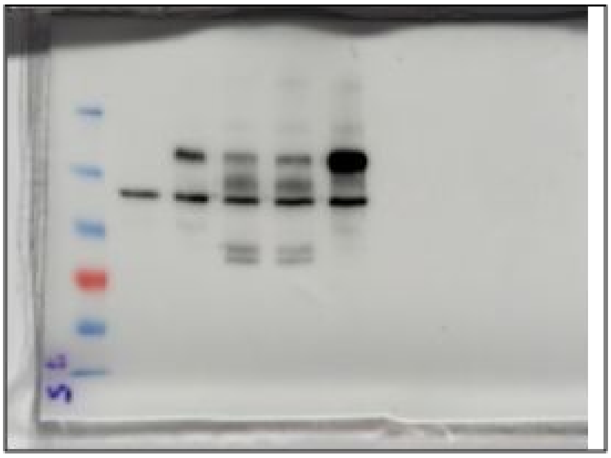

HA tag
(N-LPHN3)

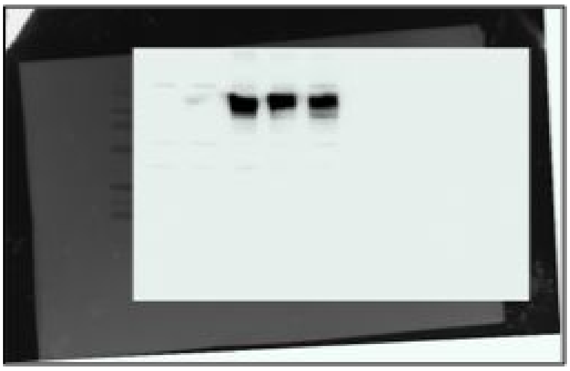

meprin β

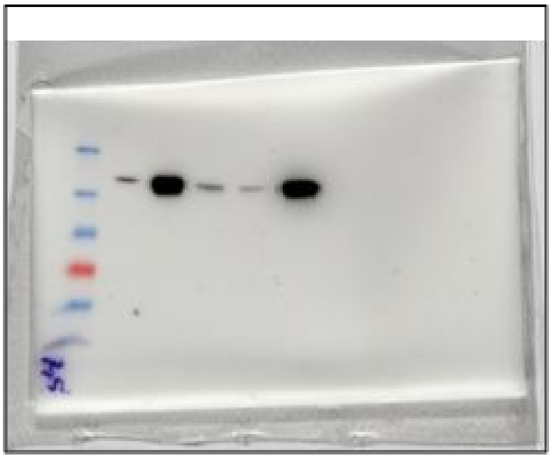

LPHN3

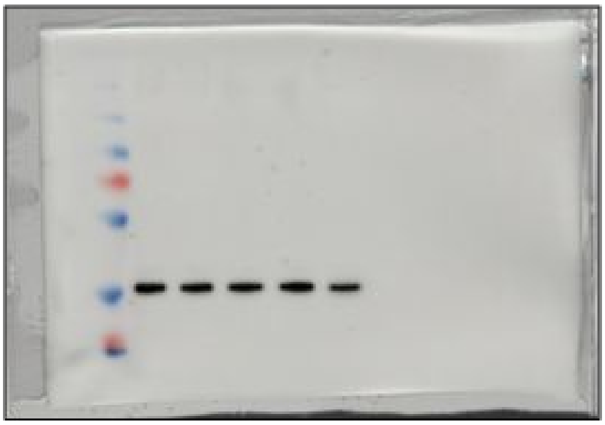

GAPDH

supernatant

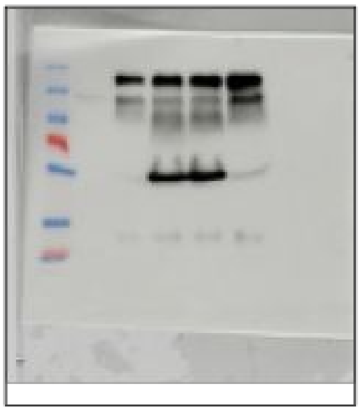

HA tag
(N-LPHN3)
